# The effect of heterogeneous environmental regulations on the employment skill structure: The system-GMM approach and mediation model

**DOI:** 10.1371/journal.pone.0290276

**Published:** 2023-08-24

**Authors:** Yuhan Jing, Mingzhao Hu, Lingdi Zhao

**Affiliations:** 1 School of Economics, Ocean University of China, Qingdao, Shandong, China; 2 Department of Statistics and Applied Probability, University of California, Santa Barbara, CA, United States of America; East China Normal University, CHINA

## Abstract

Environmental regulation has played an essential function in reducing pollution and it also influences the flow of labor. Although studies on employment and environmental regulation have gained prominence, most researches ignore the heterogeneity of regulatory tools and its discrepant impacts on different skilled labor; moreover, few literatures have explored how environmental regulations affect employment. Therefore, this study creatively incorporates environmental regulation, industrial green transformation and employment skill structure into a unified analytical framework, categorizing environmental regulations into command-and-control type, market-incentive type and voluntary type and analyzing the heterogeneous influences of environmental regulations on employment skill structure. Meanwhile, we explore the indirect impact of environmental regulations on the employment skill structure from the mediating role of industrial green transformation. The following are the research findings: (1) From a national perspective, both command-and-control and market-incentive types present a U-shaped association with employment skill structure, and their intensity has not surpassed the turning point yet; while the voluntary type is positively connected with the employment skill structure. (2) From the regional analysis, the findings in central and western areas are consistent with the national results; while the market-incentive and voluntary types show a reciprocal U-shaped connection with employment skill structure in eastern, and their regulatory intensity is in the rising stage of the curve. (3) Industrial green transformation acts as a partly mediator between market-incentive type and employment skill structure, but presents a suppression effect between command-and-control type, voluntary type, and employment skill structure. This paper takes industrial green transformation as the mediating variable, which emphasizes the importance of industrial green transformation and enhances the understanding on the mechanism of environmental regulation influencing employment skill structure. The research results provide theoretical support and significant reference for China in formulating policies to facilitate industrial green transformation, mitigate pollution, and optimize employment skill structure.

## 1. Introduction

The global climate is currently warming [1, 2], and ecological damage and environmental pollution are threatening human existence. Green and low-carbon society have become the universal development goals pursued by all countries [3]. Because of environmental degradation and resource depletion, China has had to alter its extensive industrialization process and regularly update environmental regulations to accomplish long-term development. As an essential green policy tool, environmental regulation affects the employment scale [4–6].

The current researches about the influences of environmental regulation upon livelihoods began with the employment scale effect. Many academics insist that environmental regulations suppress employment [7]. This is because the increased cost of pollution control is incorporated into the manufacture expenses of firms, and enterprises will downsize the staffs or decease the scale of production in order to maintain profits, which either directly or indirectly reduces the demand for labor [8, 9]. Others argue that environmental regulation promotes employment [10, 11]. On the one hand, an appropriate intensity of regulation will push firms to innovate technologically and improve productivity [12], leading in a growth in production scale and labor demand [13]. On the other hand, rising energy costs will compel enterprises to replace them with other production factors, such as labor, which increases the employment [14]. The remaining scholars consider that the environmental regulations’ impacts on job is inconclusive [15], and indicate that the introduction of environmental restrictions produces some jobs while destroying others, with minimal effect on the overall employment scale [16].

Although the current study has not come a unified conclusion on if environmental regulations have an advantageous influence on employment, it has been acknowledged that the employment scale is influenced by the environmental regulation [6]. Additionally, China is facing with the disappearance of the demographic dividend and other population issues [17]. Demographic problems and environmental pressures have resulted in changes in the employment skill structure: The execution of some environmental policies has increased the expenditure of emissions and even halted production for rectification, resulting in the layoff of some low-skilled workers [18]. In contrast, the growth of emerging green firms has increased the demand for highly educated and high-skilled labor. Obviously, environmental regulations have varying consequences on employees of different skill levels. So, what implications do diverse kinds of environmental regulation have upon the employment skill structure?

Chinese industrial expansion is the primary source of pollution [19]. It’s critical to promote transformation of industries towards energy savings, pollution elimination, and greening. What function does environmental regulation perform in the industrial greening transition? According to one viewpoint, total factor productivity plays a mediated function. Only when regulation intensity is moderate can total factor productivity improve, ultimately stimulating the green transition of industrial development mode [20, 21]. Others hold that technical innovation contributes to environmental regulation and the industrial green transition [22, 23]. Many studies, however, argue that the influence of regulations upon industries’ greening transition is indeterminate because of many factors such as the regulatory standard, environmental obstacles, and there may be a nonlinear relationship between the two [24, 25].

In conclusion, there are few literatures have explored the relation among environmental regulations, industrial green transformation, and job skill structure. Given this, based on the System Generalized Method of Moment (system-GMM) [26] and the mediating model, the study innovatively examines the influences of various categories of environmental regulations upon the job skill structure, as well as the mediating effect of industrial greening transition. These are the contributions of this study: Firstly, using the entropy weight approach, this paper differentiates three kinds of environmental regulation, establishes the comprehensive index system for diverse kinds of regulations and the level of industrial greening transition. Meanwhile, the article looks into the effects of command-and-control environmental regulation, market-incentive environmental regulation, and voluntary environmental regulation [27] on the employment skill structure severally. Moreover, we analyze the heterogeneity among eastern, central and western provinces, which broadens our theoretical knowledge about the relation among environmental regulations and job skill structure. Secondly, the research creatively attempts to incorporate environmental regulation, industrial green transition and job skill structure into a same analysis frame. And we adopt industrial green transition as a mediating variable to investigate the interior conduction system of environmental regulation influencing employment skill structure, which breaks the limitation of previous studies that only involved each other’s relationships. The results are a significant reference for formulating policies, promoting sustainable development, improving people’s livelihoods and stabilizing employment.

Following is the organization of the remaining sections of this research. Section 2 reviews the existing studies and put forward hypotheses; Section 3 shows the materials and methods; then, the fourth section includes the discussion of the results. At the end of this article, we make some suggestions according to the findings in section 5.

## 2. Literature review and hypothesis development

This section reviews literatures on environmental regulation, industrial green transformation and employment skill structure. We find that available literature focuses solely on the influences of environmental regulations upon job scale and ignores the differences between regulations. Therefore, the article categorizes environmental regulations into three types to explore the relation between regulation and job skill structure. Additionally, we use the industrial greening transition as the mediating factor to explore whether it may act as a bridge between environmental regulation and employment skill structure. This part finally proposes corresponding hypotheses based on the cost effect, the innovation offset effect, and the factor substitution effect.

### 2.1 Literature review

There are many researches on environmental regulation and job skill structure. Many studies believe that environmental regulations influence regional employment structure via labor mobility. Faced with stringent environmental regulation, pollution-intensive enterprises usually choose to relocate to areas with lax regulations [28], and the transfer of enterprises will inevitably drive labor flow, with low-skilled labor being more likely to lose their jobs [29]. Clean enterprises are more inclined to import green technique and innovate manufacturing modes, which leads to a high number of green jobs [30]. These jobs require employees with higher education, a relatively complete professional knowledge system, and the ability to operate environmental equipment proficiently, etc. It intensifies the replacement of low-skilled worker by high skill talent and contributes the upgrade of the employment skill structure [31–33]. Zhong also finds that as the strength of regulation increases, the demand of high-skilled labor will continue to rise, whereas the common labor will first decrease but afterwards return, showing a U-shaped line [6].

Environmental regulation is generally classified as formal (explicit) and informal (implicit) forms according to the existing literature [34]. The first mentioned could be subdivided into command-and-control type and market-incentive type [35–37]. The former refers to mandatory legislation, such as environmental preservation legislation. It is measured using two primary methodologies: One is the basic indicator method, it often includes the newly published laws and regulations [37, 38]. However, this method fails to embody the execution of regulation at local level administrative districts and differentiate the discrepancy of environmental regulatory implements. The other is the comprehensive indicator method [39]. Market-incentive type depends on measures like pollutant emission fees as well as tradable licenses to play a role [40]; It is often quantified in the literature using a single indication of sewage charges [34]. Voluntary environmental regulation comes up late. It is also called informal environmental regulation, which is usually presented in a voluntary way, including values, ethics, environmental certification, eco-labeling, and so on [41, 42]. It is not mandatory, but depends on the public’s environmental awareness [26], and it is an important complement to formal environmental regulations. Due to the different forms of citizen participation in environmental supervision, diverse scholars utilized different indicators for characterization, including the civic complaints on pollution incidents, the amount of articles about pollution in national media [36], the data of local pollution events and so on.

Different types of regulation modes have different social costs and effects. Command-and-control type necessitates the participation of several departments, including the government, and has a high social cost. Market-incentive type can be accomplished through the market mechanism at a low social cost. And voluntary type simply relies on the power of public and has lower social costs [43]. Various types of regulatory tools have different effects on employment structure. Some studies find that formal environmental regulation, will stimulate innovative activities in firms while simultaneously promoting the needs of highly skilled worker and optimizing the job skill structure [16, 33].

Numerous studies have explored the industrial greening transformation recently. Industrial greening transition bases upon the green economy, trying to maximize the economic profits of environmental resources. The measurements of industrial greening transition mainly include two methods: green total factor productivity and composite indication [44]. The former is commonly assessed by the Data envelopment analysis and its upgraded forms [20]. Existing literatures have not achieved an agreement about the influences of various kinds of regulations on industry’s greening transformation. According to some studies, on a long-term view, command and market types will stimulate businesses’ environmental performances, while the short-term impact is minimal. Moreover, environmental regulations have a diverse influence on various industries [45]. There are few literatures studying the relation between employment and industrial greening transition. Some scholars claim that the green economy provides a huge number of jobs in the United States, which helps to improve employment quality [46]. During the process of industrial green transition, some new jobs are being created and some old jobs are disappearing, and the change in the overall employment size hinges on the magnitude of both effects [47].

To sum up, although the researches on labor and environmental regulation is expanding, there are still some limitations in this area. Most papers regard environmental regulation as a whole, without taking into the heterogeneity of environmental regulatory instruments and effects, which is somewhat one-sided [36]. And most of current researches pay attention to the influences of regulation and job size, with little concern for employment skill structure. Besides, previous literatures only study the influence of industrial green transformation upon employment scale, with fewer researches investigate the relation of environmental regulations, industry green transformation and employment skill structure. Nowadays, environmental pollution and employment structure contradictions are the major obstacles that impede Chinese growth [6]. How to progress toward green development while also achieving high-quality employment is an urgent issue that must be solved at present. Given this, the article classifies environmental regulations into command-and-control type, market-incentive type and voluntary type, investigates the impact of regulations upon job skill structure, as well as the mediating role of industrial green transformation.

### 2.2 Hypothesis development

Referring to current researches, the influences of environmental regulations upon job skill structure could be categorized into three effects: First kind is the cost effect. Cost effect refers to the implementation of regulations raises the costs of pollution factors; and the increasing input in emission reduction raises the production cost of enterprises [48], crowding out part of their research and development (R&D) funds. This can squeeze out innovative activities and diminish the need for highly skilled worker. Meanwhile, businesses will transfer the costs of environmental regulations to employees through decreasing labor or lowing salaries [49], which influences the overall employment scale circuitously. In general, the cost effect is unfavorable to the upgrade of employment skill structure.

Second type is the innovation offset effect. Based on the weak Porter theories [50], tightening environmental regulations will compel businesses to upgrade product lines and processes in the long run, which raises their level of innovation and reduces pollutant emissions. From product design to production, high-skilled workers capable of technical innovation are needed. Enterprises will recruit more qualified R&D staff, which will support the optimization of regional employment skill constitution.

The third one is factor substitution effect. Implementing environmental regulations would raise the spend of resource elements for businesses, causing them to reallocate the inputs proportion of variable productive elements [14, 51]. For labor-intensive enterprises, increased environmental costs will lead to a decrease in labor input. So, many companies will lay off workers, with low-skilled labor suffering the brunt of the burden. However, environmental regulation will stimulate capital-intensive and technology-intensive businesses to buy green machines and introduce eco-friendly production technology actively, which will strongly substitute for labor and other production factors. In this process, low-skilled employees who are more mobile are more likely to be laid off [5, 29], while high-skilled labor with green technology will be more competitive [52]. As a result, the factor substitution effect promotes the upgrading of the employment skill structure.

Preceding researches show that the net effect of cost effect, innovation offset effect [6] and element substitution effect determines the influence of regulations on job skill structure. If enforcing environmental regulations makes great cost effect, it will be detrimental to the upgrading of employment skill structure. On the contrary, if the total of innovation compensation impact and factor replacement impact surpasses the cost impact, the implementation of regulations would greatly optimize job skill structure.

Command-and-control type includes government mandatory legislation [53], such as limiting the pollution emission of enterprises, shutting down and transferring highly polluting businesses. In the short run, the enforcement of it raises the manufacturing expense of businesses. Firms will spend a part of R&D funds on scale production to enhance profits rather than technological advancements [6], because technical innovation is long-periodic and has large investment expense [54]. So, the short-term implementation of it will initially inhibit the optimization of employment skill structure. At this point, the cost effect exceeds the total of factor substitution and innovation offset effects, which is unfavorable to the upgrade of employment skill constitution. However, with the intensification of regulation increases, businesses will innovate continually to minimize emission costs for long-term interests [26], which generates a large demand for high-skilled labor and ultimately facilitates the optimum and upgrade of the regional job skill constitution. Therefore, the article puts forward the hypothesis:

**H1:** Command-and-control environmental regulation will suppress the optimization of employment skill structure at first, but with the intensity of regulation increases, it will help to optimize the employment skill structure.

Market-incentive environmental regulation is primarily founded on the market mechanisms, including the carbon trading system and pollution taxes and fees. The intensity of it is small in the early stage of enforcement, and businesses will weigh the expense of violation against the innovation costs. Companies are willing to accept the penalty—paying environmental taxes or purchasing emission rights when the non-compliance costs are modest. Similar to command-and-control type, firms will use a portion of their R&D capital for mass manufacturing to offset the high expenses incurred by environmental regulations, which inhibits the innovation investment and diminishes the needs of high-skilled workforce [55]. The cost effect is visible at this time, which will be damaging to local employment skill structure upgrade. Market-incentive environmental regulation is going to play a positive function to the employment skill structure as its intensity increases. For example, taxation and subsidies for green technology R&D could stimulate businesses to pursue technological innovation [32]. At this time, the innovation offset and factor substitution effects of the regulation are adequate to compensate the cost effect [56]. Therefore, it will take a proactive part in optimizing employment skill structure in a long term. In summary, this study suggests the second hypothesis:

**H2:** Market-incentive environmental regulation is going to suppress the optimization of employment skill structure at first, but with the intensity of regulation increases, it would upgrade the employment skill structure.

Voluntary environmental regulation mostly depends on public awareness of environmental protection and conscious action by enterprises. Zheng and Shi [37] argue that citizens, communities and environmental non-governmental organizations (ONGE) are playing a more active role in improving environmental quality. In recent years, as the central environmental conservation documents released, people’s perceptions of environmental protection have been enhanced. The public is actively following and reporting on pertinent environmental issues through environmental letters and visits, as well as information medias such as the Internet and TV. This will have an influence on the reputation of polluting enterprises [36], prompting them to actively carry out eco-friendly technologies [57]. The innovation compensation and factor replacement effects will emerge gradually. Furthermore, enterprises will hire more R&D workers who master green technologies [31], which increases the need for high-skilled worker. Moreover, as workers become more environmentally concerned, they will also take the initiative to learn new green technology and continuously enhance their competitiveness. Therefore, the implementation of it will facilitate the upgrade of regional employment skill structure. In consideration of preceding studies, we come up with the assumption:

**H3:** Voluntary environmental regulation will optimize the employment skill structure.

For exploring the underlying mechanisms about the influence on various types of environmental regulations upon employment skill structure, this study for the first time attempts to take industrial green transformation as the mediating variable. The following is the mediation mechanism analysis.

Command-and-control type reduces high-polluting enterprises by shutting down and transferring high-energy-consuming factories, as well as restricting access to polluted industries [35, 36], which forces enterprises to shift towards energy saving and greening. This is beneficial to increasing the energy efficiencies and encouraging the local industrial green transformation process. During the process, industries with high energy usage and emissions are continuously optimized and upgraded, which needs more high-skilled personnel with core R&D technologies to match; while those low-skilled labor who can only engage in simple production will lose their jobs [6]. This indirectly improves the employment skill structure. Hence, the article puts forward another hypothesis:

**H4a:** Command-and-control environmental regulation could upgrade employment skill structure via industrial green transformation.

As for market-incentive type regulation, such as pollution charges, has compelled industrial enterprises to alter traditional processes and actively develop green and low-carbon technologies. Moreover, environmental conservation subsidy policy and tax incentive system provide enterprises with funds for environmental innovation and encourage them to carry out innovation activities actively [58], all of which will accelerate the local industry greening transition. The transition would spawn a number of emerging green industries. These industries have high labor demands [31] and require high-skilled workers with great comprehensive quality and capable of conducting R&D in environmental technologies particularly [30].For another, some easy duties will be automated, and those low-skilled workers who are less educated and unable to learn and master new equipment quickly will be compelled to be laid off [31]. This indirectly raises the share of high-skilled labor and supports the upgrading of regional employment skill structure [11]. Hence, hypothesis H4b is proposed:

**H4b:** Market-incentive environmental regulation would optimize employment skill structure through industrial green transformation.

The implementation of voluntary environmental regulation allows citizens to submit environmental appeals through petition, proposals and other forms [36], which prompts enterprises to prioritize the balance of environmental and economic benefits. In order to meet consumers’ preference for environment-friendly products, enterprises will manufacture green products [59]. Meanwhile, governments will also give fines and other administrative penalties for pollution enterprises in response to public complaints, further forcing enterprises to reduce pollution and emissions and accelerating the industrial green transformation. During this transition, enterprises will hire more skilled workers while reducing the number of production positions, which will have certain influence on the job skill structure. Therefore, the hypothesis H4c is proposed:

**H4c:** Voluntary environmental regulation could promote the upgrade of employment skill structure through industrial green transformation.

## 3. Materials and methods

This section discusses the variable selection, variable measurement, model settings and the data source to explore various implications of diverse environmental regulation on job skill structure and their internal mechanisms empirically. The article constructs the comprehensive index system of three types regulations as the explanatory variables respectively. Meanwhile, by creating a comprehensive index system of the industrial green transformation, this paper innovatively takes it into the empirical analysis to explore whether it can be used as a connection between environmental regulation and employment skill structure.

### 3.1 Variables selection

#### 3.1.1 Explained variable

Employment skill structure (ems1_it_): Due to the disparities in educational level and individual skills, the labor force is gradually differentiated into skillful and low-skilled labor. Considering the difficulty in obtaining data on the labor force with intermediate and advanced certificates in each province, this paper refers to Zhong’ research [6], in which workers with vocational college diplomas are regarded as high-skilled labor. And people who have completed education at high school, trade school, junior high school, elementary school or below are considered as low-skilled labor. The article utilizes the portion of high-skilled labor to measure the employment skill constitution. Moreover, environmental regulation mostly targets industrial polluting enterprises [60]. Hence, based on the classification criteria of China Industrial Statistical Yearbook, this paper selects the proportion of the labor force with a college diploma and above (ems1_it_) in mining, manufacturing, electricity, heat and fuel power and water manufacturing and provision industries to evaluate the employment skill structure. And the article adopts the percentage of R&D employees to the workforce (ems2_it_) at above-scale industrial enterprises in the aforementioned industries as an alternative variable for the robustness test.

#### 3.1.2 Independent variable

To reflect the execution of regulations, such as the intensity of law enforcement, this paper adopts the comprehensive indicator method to measure command-and-control environmental regulation (CER). Four indicators are selected: the amount of regional administrative punishment incidents [61], the amount of staffs inside the eco systems [26, 62], and the amount of environmental adoption projects. Then the article calculates the proportion of each indicator to local GDP and builds a comprehensive index system.

For market-incentive environmental regulation (MER), most of the current preferential subsidy policies are concentrated on industries, and the pollution rights trading system remains in the experimental phase, both of which lack national data. Therefore, the paper uses the amount of spending in emission controlling as the government incentive measure and the punitive measure is represented by the amount of deliver tax payments on pollutant charges, calculating it comprehensively. To be more precisely, the article calculates the proportion of regional GDP spent on environmental protection of environmental protection acceptance projects [36, 63], the proportion of expenditure on controlling industrial emission sources and the percentage of sewage discharges to regional industrial added value respectively [61, 64], and the proportion of expense in emission control project completed this year to regional GDP [61]. The comprehensive indication system is constructed.

Referring to previous studies and considering the limited access to R&D funding and patent data of industrial enterprises, this paper finally selects three indicators to measure voluntary environmental regulation (VER): the number of scientific research institutions of industrial businesses over the scale [65], letters and visits’ amount, the number of suggestions from the National People’s Congress [66] and proposals from the Chinese People’s Political Consultative Conference [26]. We construct a comprehensive indicator system to represent people’s environmental consciousness and attention to environment. The comprehensive scorings of three categories regulation computed by the entropy weight approach represent the core explanatory variables–three categories of environmental regulations.

#### 3.1.3 Mediation variable

As for industrial greening transition, Feng argues that it ought be reflected in the increased production efficiency, the reduction of environmental impact [67], the decrease of pollution emissions, and the improvement of resource utilization [26]. Therefore, combined with existing research [68], this paper measures industrial green transformation across multiple dimensions, including resource intensive utilization [69], pollution degree [70], industrial structure, and sustainable growth [71]. In addition, we calculate the weights of every indicator by using the entropy approach, establishing a comprehensive index system for measuring industry’s greening transition (ingt_it_). Among them, the six high-power-consumption industries are defined from “the National Economic and Social Development Statistics Report in 2010” [72].

From Table 1, the index attributes include both positive and negative indicators. The positive indicator indicates that it is beneficial to the industrial greening transition, whereas the negative indicators show that they are detrimental to the transformation. The first level indicators of resource intensive utilization, pollution degree and industrial structure are all negative attributes.

**Table 1:** Comprehensive indexes of industrial green transformation.

Target layer	First level index	Secondary level index	Definition	Attributes	Weight
Degree of industrial green transformation	Resource intensive utilization	Energy consumption per unit of industrial value added	Annual energy consumption / industrial value added	-	0.091
		Water consumption per unit of industrial value added	Annual industrial water consumption / industrial value added	-	0.122
	Pollution degree	SO2 emissions per unit of industrial value added	Annual industrial sulfur dioxide emissions / industrial added value	-	0.126
		CO2 emissions per unit of industrial value added	Annual carbon emission of industrial dioxide / industrial added value	-	0.039
		Wastewater discharge per unit of industrial value added	Annual discharge of industrial wastewater / industrial added value	-	0.114
	Industrial structure	The proportion of six high-energy-consuming industries	Annual added value of six energy-intensive industries / industrial added value	-	0.042
	Sustainable development	Comprehensive utilization rate of industrial solid waste	Comprehensive utilization of industrial solid waste / production	+	0.466

For example, in the pollution degree, the outflow of wastewater is a negative indicator. The greater discharge of wastewater, the worse intensive use of resources, which is harmful to the industry transition. While the sustainable development index is a positive indicator, which means that the higher the compositive usage ratio [73], the more favorable to sustainable development and is beneficial to industrial greening transition. The weight is calculated by the information supplied by each index [26]. If an indicator’s variation changes greatly, it gives more information [74]. Therefore, the comprehensive evaluation is more influenced by this index, so it is assigned a larger weight [75]. And vice versa. From Table 1, the weight of sustainable development index is more than 0.45, which has a greater impact on industrial green transformation. Therefore, enterprise should continuously improve the overall utilization rate of industrial waste so as to support the industrial greening transition and the sustainable growth. The weight of sulfur dioxide emission and wastewater discharge in the pollution degree index is 0.126 and 0.114 respectively, which is also large. This shows that enterprises should continue to reduce the discharge of waste water and exhaust gas to mitigate environmental pollution. The weights of resource intensive utilization and industrial structure are minimal, indicating that the change amplitude of these indicators is relatively stable.

#### 3.1.4 Controlled variables

This article controls these variables:

The degree of foreign capital utilization (fdi_it_): Foreign capital will alter the industrial structure, which will in turn affect the employment skill structure to some extent. When foreign capital flows into labor-intensive industries, it will motivate the employment of low-skilled worker [36]; however, when it flows into technology-intensive industries, it will stimulate the need of skillful workers, affecting the regional job skill construction eventually [26]. fdi_it_ denotes the proportion of foreign direct investments to GDP.

Educational attainment (edu_it_): Enhancing the level of education improves employees’ ability to acquire new technologies, which upgrades the regional employment skill structure [33]. The article adopts the share of students in normal university to the entire regional people to represent edu_it_ [26].

Level of technological innovation (R&D_it_) may affect the employment structure [56, 76]. This is due to the fact that technological advancement would increase the demand for R&D innovative talents, which is conducive to promoting an advanced employment skill structure. R&D_it_ is defined as the percentage of R&D expense to GDP for above-scale industrial businesses.

Capital stock (k_it_): An increase in capital stock implies deeper capital deepening, and more funds are available for industry operation and expansion, which affects the demand of enterprises for different skilled labor. The amount of fixed capital formation is measured by the perpetual inventory approach [77].

Wage level (lnwage_it_) is significant for employment, and rising wages will not only increase employees’ willingness to work, but also motivate workers to learn new skills. The article adopts the average wages of urban workers in mining, manufacturing, electricity, heat and fuel power and water production and supply industries [78] to represent lnwage_it_, and takes the average salary in 2000 as the basic period for price deflator.

### 3.2 Research method

The following are the key research methods used in the article: First, the comprehensive scores of three types regulations as well as the degree of industrial greening transition are calculated using the entropy approach. Second, by introducing the lag term of employment skill structure, the basic regression model is established using the system-GMM model. Third, we investigate the mediating role of industrial greening transition by the mediation model.

#### 3.2.1 The entropy value method

The entropy approach takes the difference between data as the main benchmark, which is more objective and more accurate, hence, this study takes the entropy weighting approach to assess three environmental regulations’ levels and the degree of industrial greening transformation [65, 79]. Following are the procedures for calculating:

**Step 1:** Data should be standardized:

positive indicator:

$$X_{ij}=\frac{x_{ij}-min(x_{ij})}{\max\left(x_{ij}\right)-min(x_{ij})}$$ (1)

negative indicator:

$$X_{ij}=\frac{max(x_{ij}){-x}_{ij}}{\max\left(x_{ij}\right)-min(x_{ij})}$$ (2)

Among them, x_ij_ represents the indication j’ data of a province in the year i, X_ij_ denotes the standardized data for indicator j of a province in year i, max (x_ij_) and min (x_ij_) indicate the maximum and minimum values of the indicator respectively.

**Step 2:** Calculate the ratio of the first index.

$$P_{ij}=\frac{X_{ij}}{\sum_{i=1}^{m}X_{ij}}$$ (3)

**Step 3:** Define the entropy value of the j index.

$$e_{j}=-\frac{1}{lnm}\sum_{i=1}^{m}P_{ij}ln(P_{ij})$$ (4)

$$g_{j}=\frac{1-e_{j}}{n-\sum_{j=1}^{n}e_{j}}$$ (5)

Where 0<g_j_<1,

**Step 4:** Calculate the index weight.

$$w_{j}=\frac{g_{j}}{\sum_{j=1}^{n}g_{j}}$$ (6)

**Step 5:** Calculate overall score.


$$S_{j}=\sum_{j=1}^{n}w_{j}P_{ij}$$ (7)

#### 3.2.2 Basic regression model—dynamic panel

According to the New Keynesian hysteresis theory of unemployment lag [80, 81], the employment equilibrium is connected to the lag of the real employment rate. Therefore, the present employment is greatly influenced by the preceding period. In order to more accurately estimate the impacts of regulations on the employment skill structure, the paper incorporates a lagged term of the employment skill structure. However, this may result in a correlation among explaining variables and random error terms. The article adopts the system-GMM approach for regression analysis to address the aforementioned challenges and estimate accurately, which is more efficient in the estimation [82, 83]. Following are the brief description of system-GMM.

$$y_{\mathrm{i,t}}=\alpha_{0}+\alpha_{1}y_{\mathrm{i,t}‐1}+\beta X_{i,t}+\varepsilon_{\mathrm{it}}$$ (8)

Formula (8) is the basic dynamic panel model. Where y_i,t_ is the dependent variable, y_i,t-1_ is the lag term, *X*_i,t_ is the explanatory variable, and ε_it_ is the random error term. First, we apply a first-order difference to the equation:

$$\Delta y_{\mathrm{i,t}}=\alpha_{1}\Delta y_{\mathrm{i,t}‐1}+\Delta \beta X_{i,t}+{\Delta \varepsilon }_{\mathrm{it}}$$ (9)

At this point, ε_it_ is related to Δy_i,t-1_. Under the assumption of no autocorrelation, y_i,t-2_ is not related to Δε_it_ and Δy_i,t-1_, as a result, y_i,t-2_ is capable of being the instrumental variable of y_i,t-1_. This is the use of lag variables as tool variables for estimation—difference GMM estimation. Whenever y_i,t_ is near to a random walk in differential estimation [84], y_i,t-2_ becomes a weak instrumental variable. Therefore, we use Δy_i,t-1_ as the instrumental variable of y_i,t-1_ for the level equation [84]. System-GMM integrates the distinction equation with the level equation for regression, reducing the estimation bias even more. The lagged term of employment skill structure is used as the instrumental variable in the article, which not only reduces the endogenous influence as far as possible, but also overcomes the heteroscedasticity and the bias induced by serial correlation [85]. In addition, in order to verify the relation among regulations and employment skill structure accurately, the article adds a quadratic term of regulations. We also use relevant variables’ logarithms to decrease heteroscedasticities’ impact [26]. The benchmark model is constructed as follows:

$${ems}_{\mathrm{it}}=\alpha_{0}+\alpha_{1}{ems}_{\mathrm{i,t}‐1}+\alpha_{2}{CER}_{\mathrm{i,t}}+\alpha_{3}{\left({CER}_{\mathrm{i,t}}\right)}^{2}+\alpha_{4}{Control}_{it}+\varepsilon_{\mathrm{it}}$$ (10)

$${ems}_{\mathrm{it}}=\alpha_{0}+\alpha_{1}{ems}_{\mathrm{i,t}‐1}+\alpha_{2}{MER}_{\mathrm{i,t}}+\alpha_{3}{\left({MER}_{\mathrm{i,t}}\right)}^{2}+\alpha_{4}{Control}_{it}+\varepsilon_{\mathrm{it}}$$ (11)

$${ems}_{\mathrm{it}}=\alpha_{0}+\alpha_{1}{ems}_{\mathrm{i,t}‐1}+\alpha_{2}{VER}_{\mathrm{i,t}}+\alpha_{3}{\left({VER}_{\mathrm{i,t}}\right)}^{2}+\alpha_{4}{Control}_{it}+\varepsilon_{\mathrm{it}}$$ (12)

Where i indicates the province, t is the year, ems_it_ represents the employment skill structure of i province in year t, ems_i,t-1_ represents the employment skill structure level of i province in previous phase; α_0_ is the intercept term; CER_i,t_, MER_i,t_, and VER_i,t_ represents three types of environmental regulations respectively; control indicates controlling variables, including the degree of foreign capital utilization (fdi_it_), educational qualifications (edu_it_), the degree of capital deepening (k_it_), the regional wage level (lnwage_it_), and the degree of technical innovation (R&D_it_); ε_it_ is the random error term.

#### 3.2.3 The mediating effect model

Environmental regulations affect employment as well as the industrial greening transformation [86]. During the process of transformation, there could be several old jobs removed and new positions created. Given the scarcity of literatures about the relation upon regulation, job skill structure, as well as industrial greening transformation, this article creatively takes the industrial greening transition as a mediating variable, setting up the mediating effect model to explore.

The mediation effect is that the independent variable influences the dependent variable via the mediating variable [87]. Based on Kenny and Mac [88, 89], we choose the stepwise regression approach to explore the mediation impact. To examine if diverse varieties of environmental regulations could influence employment skill structure via industrial green transformation, this article constructs Eqs (10)–(12) to discuss the gross impacts of diverse regulations upon job skill structure respectively, which are mirrored in the coefficient (α_2_) and (α_3_). Secondly, we incorporate the square term of CER, MER, VER and constructs Eqs (13)–(15) to examine the impacts of diverse categories of regulation upon the greening transformation and verify the possible nonlinear link between them. Coefficients (β_2_) and (β_3_) denote the impact of environmental regulations upon industrial greening transformation. Finally, models (16)-(18) are constructed to explore the effect of three kinds regulation as well as industry greening transformation on employment skill structure severally. Where coefficient (γ_2_) and (γ_3_) represents the immediate influence of different environmental regulations upon job skill structure; the coefficient (γ_4_) indicates the indirect influence of regulations upon job skill structure via industrial green transformation.

$${ingt}_{\mathrm{it}}=\beta_{0}+\beta_{1}{ingt}_{\mathrm{i,t}‐1}+\beta_{2}{CER}_{\mathrm{i,t}}+\beta_{3}{\left({CER}_{\mathrm{i,t}}\right)}^{2}+\beta_{4}{Control}_{it}+\varepsilon_{\mathrm{it}}$$ (13)

$${ingt}_{\mathrm{it}}=\beta_{0}+\beta_{1}{ingt}_{\mathrm{i,t}‐1}+\beta_{2}{MER}_{\mathrm{i,t}}+\beta_{3}{\left({MER}_{\mathrm{i,t}}\right)}^{2}+\beta_{4}{Control}_{it}+\varepsilon_{\mathrm{it}}$$ (14)

$${ingt}_{\mathrm{it}}=\beta_{0}+\beta_{1}{ingt}_{\mathrm{i,t}‐1}+\beta_{2}{VER}_{\mathrm{i,t}}+\beta_{3}{\left({VER}_{\mathrm{i,t}}\right)}^{2}+\beta_{4}{Control}_{it}+\varepsilon_{\mathrm{it}}$$ (15)

$${ems}_{\mathrm{it}}=\gamma_{0}+\gamma_{1}{ems}_{\mathrm{i,t}‐1}+\gamma_{2}{CER}_{\mathrm{i,t}}+\gamma_{3}{\left({CER}_{\mathrm{i,t}}\right)}^{2}+\gamma_{4}{ingt}_{\mathrm{it}}+\gamma_{5}{Control}_{it}+\varepsilon_{\mathrm{it}}$$ (16)

$${ems}_{\mathrm{it}}=\gamma_{0}+\gamma_{1}{ems}_{\mathrm{i,t}‐1}+\gamma_{2}{CER}_{\mathrm{i,t}}+\gamma_{3}{\left({CER}_{\mathrm{i,t}}\right)}^{2}+\gamma_{4}{ingt}_{\mathrm{it}}+\gamma_{5}{Control}_{it}+\varepsilon_{\mathrm{it}}$$ (17)

$${ems}_{\mathrm{it}}=\gamma_{0}+\gamma_{1}{ems}_{\mathrm{i,t}‐1}+\gamma_{2}{CER}_{\mathrm{i,t}}+\gamma_{3}{\left({CER}_{\mathrm{i,t}}\right)}^{2}+\gamma_{4}{ingt}_{\mathrm{it}}+\gamma_{5}{Control}_{it}+\varepsilon_{\mathrm{it}}$$ (18)

According to the test of mediating effect: first, check whether the coefficient (α_2_) is significant, and then determine the significance of the coefficient (β_2_) and (γ_4_) under the premise that (α_2_) is significant; if coefficient (β_2_) and (γ_4_) are all significant, then test whether the factor (γ_2_) is significant. If so, there is a sectional mediation impact [90]. If at least one of the coefficients (β_2_) and (γ_4_) is insignificant, according to the judgment method of Mac on the mediation and suppression effects [89], we adapt the Bootstrap test [91] to determine the final effects.

Bootstrap is a method of taking repeated samples from a sample. Referring to Wen [92], this paper adopted bootstrap sampling approach to determine if the indirect influence is 0 (H0: β_2_*γ_4_ = 0). The sampling times are generally at least 1000 times. By sampling 1000 times, we can get 1000 product estimates of the coefficients {$\hat{\beta_{2}}*\hat{\gamma_{4}}$}; the 2.5 percentile and the 97.5 percentile constitute a 95% confidence interval for (β_2_*γ_4_). According to this, if the confidence interval excludes 0, indicating that the product of the coefficients is significant. The confidence interval calculated through Bootstrap is more accurate and has higher testing power, which is a recognized method for directly testing the product of coefficients.

#### 3.2.4 Sample and data

Since 2000, China has put forward the tenth Five-Year Plan and specified unequivocally that the acid rain control zones and the sulfur dioxide pollution control zones should be strictly enforced [93], which are the representative instances of environmental regulation [94]. Since the year 2000, environmental regulations have played an essential part in lowing pollutants. Additionally, a lot of data has not been published since 2020, which makes it impossible to conduct a complete empirical analysis. Therefore, the article adopts the data of 30 Chinese provinces and municipalities from 2000 to 2019 (exclude Tibet, Taiwan, Hong Kong, and Macao), which from China Statistical Yearbook, China Environmental Yearbook, China Environmental Statistical Yearbook, China Industrial Statistical Yearbook, China Population and Employment Statistical Yearbook, China Urban Statistical Yearbook, Provincial and Municipal Statistical Yearbooks and Statistical Bulletins, and the interpolation approach is used to fill in the lacking data [26].

At the end, the article adopts the natural logarithm method to exclude the potential heteroscedasticity [95]. Table 2 is the descriptive statistical analysis of all variables. The large discrepancy between the maximum and minimum values of the employment skill structure indicates that the proportion of high-skilled labor is constantly changing during the sample period, and the difference between areas is significant. The mean values of command-and-control type, market-incentive type, and voluntary type regulations are 0.12, 0.126 and 0.0623 respectively, with maximum values of 0.594,0.747 and 0.59. This highlights the significant disparities in the intensity of each regulation instrument, among which voluntary type’s average intensity is the lowest; meanwhile, the change range in intensity of each regulatory instrument is relatively large. It shows that the regulatory intensity of three regulatory fools changes greatly across China, which lays the groundwork for further investigation into the diverse effect of regulations on employment skill structure. The mean number of industrial green transformation is 0.645, the minimum value is 0.274, and the standard deviation is large, implying that the transformation degree varies greatly among provinces; by analyzing the control variables, we also discover that the maximum value of each variable differs significantly. As a result, the regional heterogeneity study is required.

**Table 2:** Descriptive statistical analysis of variables.

Variable	Definition	Obs.	Mean	Std.Dev.	Min.	Max.
ems1_it_	The proportion of labor with a college diploma and above	600	0.130	0.0953	0.0078	0.622
ems2_it_	The percentage of R&D employees to the workforce	600	0.0301	0.0142	0.0047	0.113
CER_i,t_	Command-and-control environmental regulation	600	0.120	0.0833	0.0116	0.594
MER_i,t_	Market-incentive environmental regulation	600	0.126	0.0751	0.0113	0.747
VER_i,t_	Voluntary environmental regulation	600	0.0623	0.0828	0.0065	0.590
ingt_it_	Industrial green transformation	600	0.645	0.122	0.274	0.849
fdi_it_	The degree of foreign capital utilization	600	0.0631	0.0717	0.0066	0.763
edu_it_	Educational attainment	600	0.0155	0.0072	0.0021	0.0357
k_it_	Capital stock	600	9.933	0.984	7.359	11.99
R&D_it_	Level of technological innovation	600	0.937	2.394	0.0008	38.75
lnwage_it_	Wage level	600	9.310	0.290	8.843	10.35

## 4 Results and discussion

According to the models in section 3, this section undertakes an empirical examination. To begin, this part analyzes the calculation results of three types regulations and industrial greening transition in time and space. And then we discuss the basic regression results. Moreover, this section makes the regional heterogeneity analysis and mediating effect analysis. At last, the article also tests the robustness to increase the results’ accuracy.

### 4.1 Results

#### 4.1.1 Calculation results of environmental regulations and industrial green transformation

**Fig 1:**
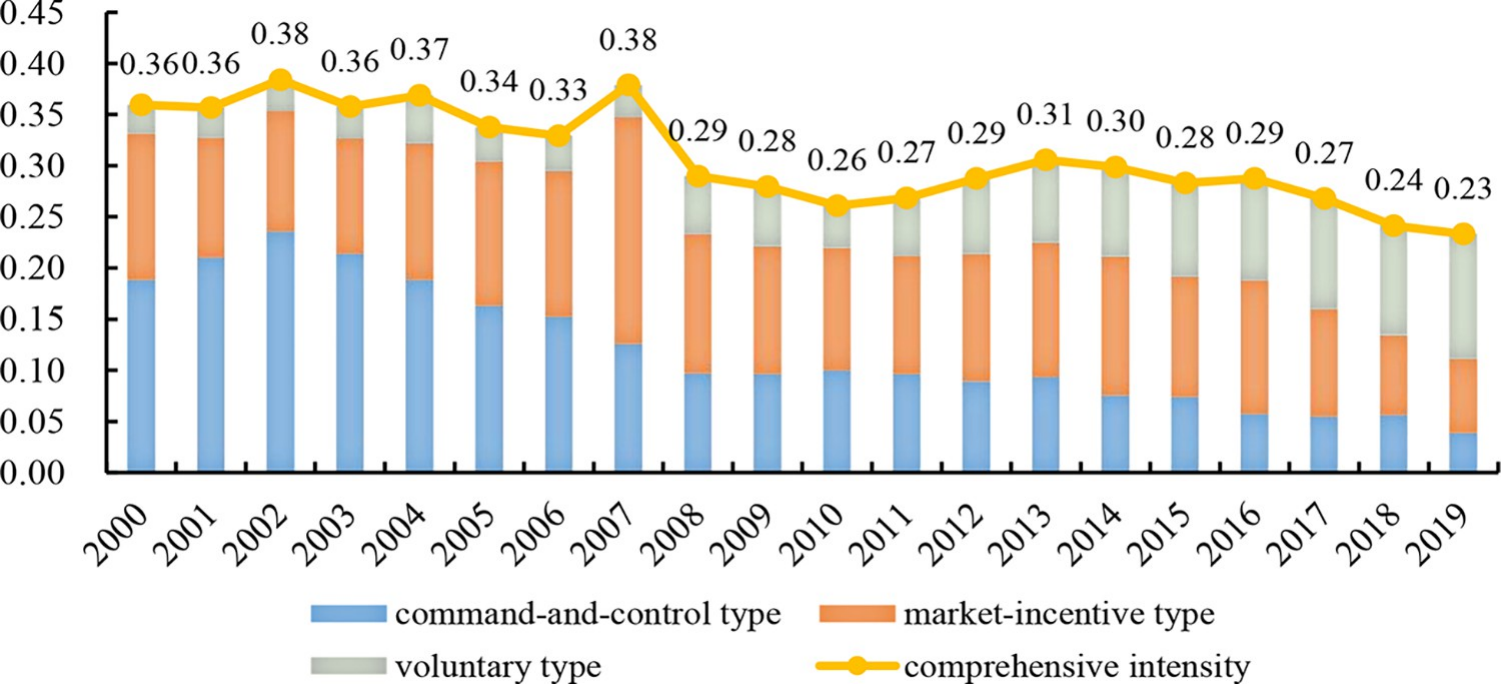
The trend on the intensity of environmental regulations in China from 2000 to 2019.

Fig 1 shows the overall comprehensive regulation intensification, which first strengthened, then weakened, and finally remained stable during 2000 to 2019 in China. It is the same as the development process of Chinese environmental regulations, which blindly increased the intension of environmental regulations in early stage, followed by adjustments, and gradually matured. For the three kinds of regulations, market-incentive type is relatively stable, while the intensity of command-and-control type gradually decreases by about 79.6 percentage points; in contrast, the intensity of voluntary type increases by a factor of 4.3. This is mainly due to the fact that command-and-control type was widely used at the early stage of regulation to achieve considerable environmental governance effects in a short time. But as the intensity of regulation has become stringent, it progressively exposed its shortcomings of high cost and low flexibility, and its main position was gradually replaced by another two types. The imbalance of localized economic growth might impede the implementation of environmental policies [96]. Given the inter-provincial developmental disparities, the article calculates the average scores of regulations in each province from 2000 to 2019 and classifies them as the eastern, central and western regions.

Table 3 demonstrates that there are indeed significant heterogeneities in the intensity across China. The regulatory intensity in eastern is significantly larger than other areas. For command-and-control type, western areas are on par whit eastern areas, and they are all significantly bigger than the middle region. This might be because the eastern area, as a pioneer area for economic development, has a higher standard for environmental protection, so the intensity of command-and-control type has been maintained at a high standard. In addition, since the western ecosystem is fragile and difficult to self-repair, the western region places a high priority on protecting the provincial ecological environments, result in augmenting regulatory strength. The scores of market-incentive type for the middle and western areas are all slightly larger than east areas, even though both are smaller than the strength of command-and-control type. This is due to the threats of industrial pollution to the environment and the vulnerability of ecosystems in the middle and western areas, which necessitates rigorous regulations, including market-incentive type. The scores of market-incentive type for the middle and western areas are all slightly larger than east areas, even though both are smaller than the strength of command-and-control type. This is due to the threats of industrial pollution to the environment and the vulnerability of ecosystems in the middle and western areas, which necessitates rigorous regulations, including market-incentive type.

**Table 3:** Intensity of three types environmental regulations in each province.

Eastern China				Central China				Western China			
Province	CER	MER	VER	Province	CER	MER	VER	Province	CER	MER	VER
Beijing	0.060	0.110	0.077	Shanxi	0.154	0.237	0.028	Inner-Mongolia	0.120	0.218	0.029
Tianjin	0.075	0.126	0.092	Jilin	0.145	0.119	0.023	Guangxi	0.132	0.120	0.026
Hebei	0.141	0.136	0.034	Heilongjiang	0.160	0.100	0.020	Chongqing	0.117	0.111	0.066
Liaoning	0.256	0.136	0.041	Anhui	0.074	0.105	0.078	Sichuan	0.093	0.083	0.039
Shanghai	0.053	0.068	0.079	Jiangxi	0.084	0.094	0.049	Guizhou	0.143	0.151	0.022
Jiangsu	0.138	0.095	0.258	Henan	0.120	0.075	0.038	Yunnan	0.121	0.111	0.030
Zhejiang	0.143	0.098	0.236	Hubei	0.081	0.083	0.046	Shaanxi	0.109	0.120	0.038
Fujian	0.123	0.085	0.076	Hunan	0.091	0.079	0.077	Gansu	0.128	0.167	0.025
Shandong	0.094	0.122	0.062					Qinghai	0.113	0.153	0.037
Guangdong	0.177	0.059	0.130					Ningxia	0.135	0.332	0.063
Hainan	0.085	0.112	0.023					Xinjiang	0.143	0.182	0.028
Average	0.122	0.104	0.101		0.114	0.111	0.045		0.123	0.159	0.037
Total	0.327				0.270				0.319		

Meanwhile, in contrast to the other areas, which concentrate on maintaining economic growth, eastern areas have a strong economic foundation and a great desire of environmental protection. Therefore, the eastern region is dominated by more stringent regulations, including command-and-control type. For voluntary type, eastern areas scored the highest, while the western areas scored the smallest, but both of their scores are smaller than the other two kinds of regulation. On the one hand, voluntary type emerges late and remains in the primary phase of development, therefore, formal regulations continue the most widely used means in China. For another, as for the standard of economic progress, eastern provinces are the developed region in China, with a high civic awareness of environmental protection as well as more comprehensive channels for environmental supervision and complaints. In addition, because voluntary type regulation is cost-effective and more flexible, the eastern region has continued to increasing its intensity.

**Fig 2:**
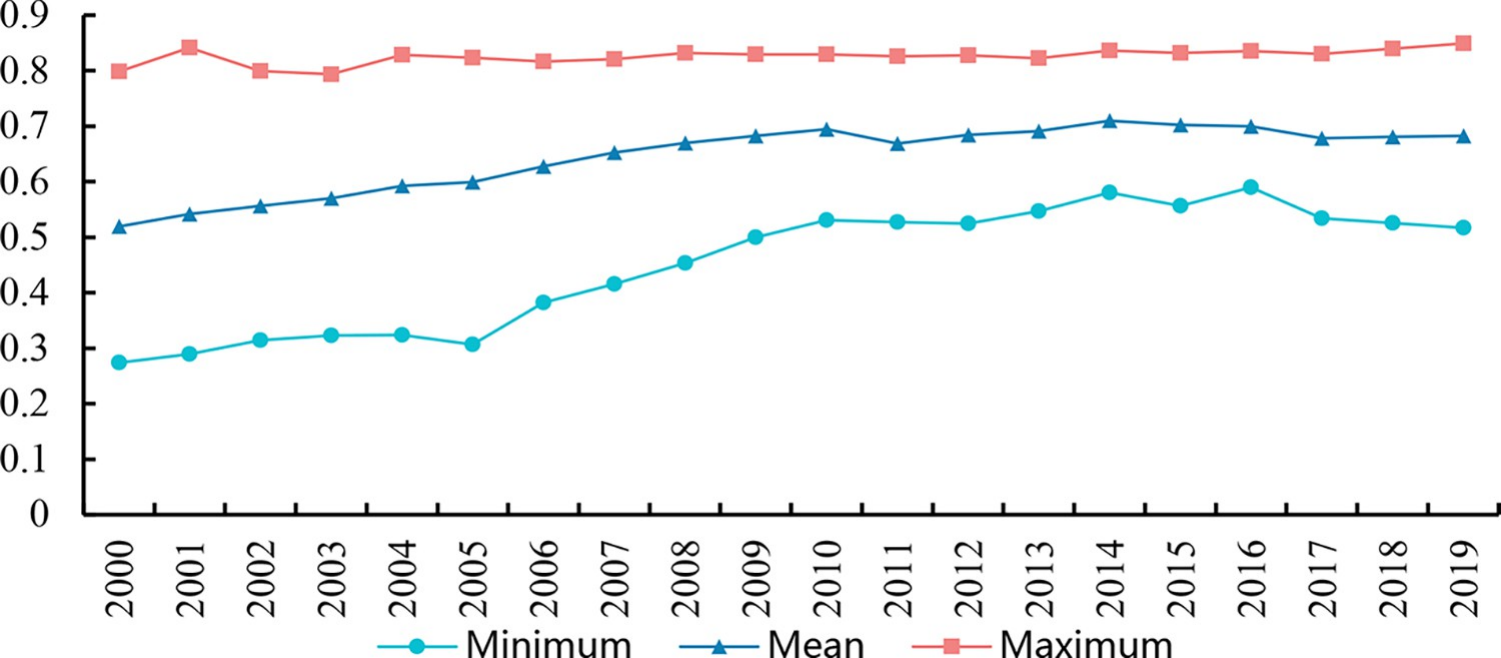
Changing trend on the level of industrial greening transition from 2000 to 2019.

Fig 2 demonstrates that the level of industrial greening transition has steadily improved in China, growing by 33.4% between 2000 and 2019. As a whole, the differences between regional industrial greening transition levels are narrowing. This indicates that thanks to the national policy deployment, the degree of industrial green transformation in various regions has been improved to different degrees. It should be noted that after 2016, the gaps widen somewhat, so the problem of imbalanced growth of industrial green transformation still needs great attention.

This study also calculates the mean value of industrial greening transformation degree for each province from 2000 to 2019 in Table 4. From Table 4, there are clear disparities in the degree of industrial greening transition in China, showing a gradually declining tendency from east to west. This is owing to the discrepancies in system, resource endowment, and economic growth level between the eastern, central, and western areas [96].

**Table 4:** Reginal average comprehensive index of industrial green transformation, 2000–2019.

Eastern China		Central China		Western China	
Province	comprehensive index	Province	comprehensive index	Province	comprehensive index
Beijing	0.738	Shanxi	0.586	Inner Mongolia	0.520
Tianjin	0.825	Jilin	0.616	Guangxi	0.568
Hebei	0.610	Heilongjiang	0.676	Chongqing	0.683
Liaoning	0.579	Anhui	0.711	Sichuan	0.582
Shanghai	0.807	Jiangxi	0.545	Guizhou	0.510
Jiangsu	0.791	Henan	0.695	Yunnan	0.575
Zhejiang	0.790	Hubei	0.676	Shaanxi	0.587
Fujian	0.700	Hunan	0.695	Gansu	0.515
Shandong	0.781			Qinghai	0.500
Guangdong	0.771			Ningxia	0.514
Hainan	0.651			Xinjiang	0.593
**Average**	0.731		0.646		0.559

#### 4.1.2 The influence of environmental regulations on employment skill structure

To prevent the pseudo-regression issue, each variable’s stationarity must be examined. There are many ways to test the unit root. For example, Li uses the Augmented Dickey-Fuller Test to test the unit root in studying housing prices’ impact [97]. We adopt the IPS test and LLC test in the article.

The test formulas are as follows:

LLC test: The null hypothesis is *δ* = 0, and the alternative hypothesis is *δ*<0. If the null hypothesis is rejected, then the unit root does not exist [98, 99].

$$\Delta y_{it}=\delta y_{i,t-1}+\sum_{L=1}^{P_{i}}\theta_{iL}\Delta y_{it-L}+\alpha_{mi}d_{mt}+\varepsilon_{it},m=1,2,3.$$ (19)

IPS test: Where *ε*_*it*_ is the white noise error term and Δ*y*_*it*_ = *y*_*it*_−*y*_*i*,*t*−1_.The null hypothesis is *β*_*i*_ = 0, and the alternative hypothesis is *β*_*i*_<0. If the null hypothesis is rejected, then the identity root does not exist [99, 100]. Table 5 is the test results.


$$\Delta y_{it}=\alpha_{i}+\beta_{i}y_{i,t-1}+\varepsilon_{it}$$ (20)

**Table 5:** Results of unit root test.

Variable	LLC test	IPS test	Stationarity
ems1_it_	-2.570***	-12.282***	Yes
ems2_it_	-5.476***	-7.535***	Yes
CER_i,t_	-4.293***	-3.169***	Yes
MER_i,t_	-3.471***	-2.903**	Yes
VER_i,t_	-9.251***	-10.909***	Yes
ingt_it_	-6.875***	-9.978***	Yes
fdi_it_	-3.649***	-2.754***	Yes
edu_it_	-3.233***	-1.986**	Yes
k_it_	-7.011***	-6.641***	Yes
R&D_it_	-5.304***	-7.303***	Yes
lnwage_it_	-4.157***	-3.225***	Yes

Note: ***, **, and * represent statistical significance at the 1%, 5%, and 10% levels, respectively.

Each variable is significant at the 5% level, denoting that the original level series is steady and suitable for regression analysis. Table 6 demonstrates that Sargan’s p-values are all above 0.7, indicating that instrumental factors do not have over-identification problems; the P values of AR (1) and AR (2) denoting that there is only first-order autocorrelation in the estimators. So, the instrumental variable is resultful and the estimated results of system-GMM are reliable. Columns (1), (3) and (5) of Table 6 are the regression results of the primary terms of three environmental regulations and employment skill structure; and columns (2), (4) and (6) are the results of incorporating the quadratic terms on this premise. Each coefficient of ems_i,t-1_ is considerably positive, suggesting that the preceding phase affects the present employment skill structure, which implies that the settings of the dynamic model are accurate.

**Table 6:** Basic regression results for the whole sample.

Variable	(1)	(2)	(3)	(4)	(5)	(6)
ems1_i,t-1_	0.939***	0.888***	0.948***	0.941***	0.970***	0.971***
	(0.011)	(0.017)	(0.013)	(0.013)	(0.013)	(0.014)
CER_i,t_	-0.055***	-0.297***				
	(0.011)	(0.044)				
CER_i,t_^2^		0.517***				
		(0.088)				
MER_i,t_			-0.067***	-0.166***		
			(0.007)	(0.034)		
MER_i,t_^2^				0.200***		
				(0.069)		
VER_i,t_					0.069***	0.064**
					(0.018)	(0.026)
VER_i,t_^2^						0.001
						(0.061)
fdi_it_	-0.029***	-0.035***	-0.021**	-0.024**	-0.058***	-0.057***
	(0.008)	(0.011)	(0.009)	(0.011)	(0.018)	(0.018)
edu_it_	0.448*	-0.146	0.869***	0.974***	1.191***	1.200***
	(0.264)	(0.257)	(0.233)	(0.185)	(0.096)	(0.111)
k_it_	0.004***	0.003***	0.004***	0.004***	0.006***	0.006***
	(0.001)	(0.0007)	(0.0007)	(0.0007)	(0.0007)	(0.0007)
R&D_it_	-0.0002	0.00007	-0.0004	-0.0004	-0.0004	-0.0003
	(0.0004)	(0.0004)	(0.0004)	(0.0003)	(0.0002)	(0.0003)
lnwage_it_	0.018**	0.035***	0.010	0.009	-0.007	-0.008
	(0.007)	(0.008)	(0.010)	(0.009)	(0.004)	(0.005)
Constant	-0.192***	-0.309***	-0.120	-0.099	0.002	0.010
	(0.068)	(0.077)	(0.092)	(0.085)	(0.042)	(0.047)
P-AR(1)	0.0002	0.0002	0.0002	0.0002	0.0002	0.0002
P-AR(2)	0.6215	0.5391	0.9694	0.9174	0.9697	0.9606
P-Sargan	1.0000	1.0000	1.0000	1.0000	0.7384	0.7428
Observations	570	570	570	570	570	570

Notes: Robust t–statistics are in parentheses

***, **, and * refer to the significance levels of 1%, 5% and 10%, respectively.

Among them, CER and MER’s coefficients of essential term and quadratic term are notably negative and positive respectively. These two kinds of regulation get a U-shaped relation to employment skill structure respectively, suggesting that the promoting effect of the two kinds regulations upon the employment skill structure do have a threshold value. Only after the intension of them surpasses a particular value will the increase in intensity optimize the job skill structure, scilicet, the performance of command-and-control and market-incentive types will suppress the optimization of employment skill structure at first; however, when the intensification enhances to a certain extent, they will optimize the employment skill structure. Hypothesis 1 and Hypothesis 2 are validated.

This is consistent with Huang’s discovery that environmental regulations would first inhibit and then increase employment [101]. The reason for this might be because, in the beginning phase of regulation, businesses frequently prefer to accept punishment [102] and respond to environmental taxes by reducing employment in the face of mandatory orders and penalties. Since the intension of command-and-control and market-incentive type at this stage is low, as a result, the penalty fines are usually less than the expense of pollution control. In this situation, the cost impact overweighs the innovation offset impact, going against to the optimization of employment skill constitution. However, as regulatory intensity increases in a long term, companies will measure the amount of penalties and the cost of optimizing polluting equipment. For the sake of long-term benefits, companies will continue to upgrade green manufacturing processes to minimize emissions costs, which necessitates a large number of high-skilled labor to match. At this time, the innovation offset effect plays an essential part in optimizing the employment skill structure.

The primary coefficient of VER is positive and significant at the 1% level, while the quadratic term of VER is insignificant. Accordingly, the relation among voluntary type and job skill structure is linear and it is positively associated with the job skill structure. Hypothesis H3 is tested. This is in line with the conclusion of Zhong. He used the amount of environmental protection recommendations to estimate the intensity of environmental regulations [6]. Referring to him, we include another two indicators, constructing a more composite indicator system of voluntary environmental regulation: the number of scientific research institutions of industrial businesses over the scale [65] and letters and visits’ amount, which enhances the validity of the estimates. The findings denote that raising the strength of voluntary type will optimize employment skill structure. This is because green and low-carbon economy has gained popularity in recent years. For one thing, governments have fully guaranteed the civic right to know and voice their concerns about the environment problems, while also constantly promoting environmental publicity and education and opening up channels for the public to appeal to environmental issues [37], which fully encourages public to participate in pro-environment and utilizes the power of environmental supervision by the masses. Faced with the environmental pressure brought by the public and media, corporate environmental expenses will grow. As a result, enterprises have to enhance technological innovation, utilize clean equipment and technology, and shift towards low-pollution and low-energy-consumption at this time. This process will create a new round of jobs. Simultaneously, in order to improve their competitiveness, workers will consciously and proactively learn new technologies and processes to improve their own skills, promoting the upgrading of regional employment skill structure. Therefore, governments should strengthen the intensity of voluntary regulation, constantly raise citizens’ awareness of environmental protection, and fully use citizens’ supervisory role in environmental governance.

As for the controlling variables, the foreign capital utilization degree (fdi_it_) of the three kinds of regulations has a considerable detrimental impact on job skill structure (ems_1it_); the coefficient of public education level (edu_it_) and capital stock (k_it_) are significantly positive. The influence of technological innovation level (R&D_it_) and wage level (lnwage_it_) is almost insignificant. It shows that at this stage, higher education level and the increased capital stock will significantly optimize the job skill structure, while increasing foreign immediate investment is detrimental to the employment skill structure.

In summary, diverse regulations have a heterogeneous influence upon employment skill structure. By dividing environmental regulations into three categories, the study discusses and expands on researches about the effect of environmental regulation upon job skill structure. Moreover, the regulatory intensity of eastern areas scored the highest at about 0.101; while the scoring of central and western areas is about 0.04. There are significant discrepancies in regulation strength between areas. As a result, this article conducts a heterogeneity analysis to provide a theoretical foundation for enacting distinguishing policy [101], which will be discussed in detail below.

### 4.2 Heterogeneity analysis

From Table 3, in comparison to the central and western areas, east area has much greater comprehensive scores for each type of regulations. This is due to the discrepancies among economic development level, inhabitants’ notion upon environmental protection and supervision channels. As a result, the research finally separates China into two samples: the eastern region, and the central and western areas to investigate the regional differential impacts [103]. Since GMM is an estimation method based on large samples, the estimation results are significantly biased whenever the sample capacity is tiny. Thus, the Bias-corrected Least Square Dummy Variable (LSDV) approach is adopted to compensate for this deficiency and improve the accuracy of the findings in this paper [104, 105].

Table 7 presents the outcomes of the regression. Command-and-control type and employment skill structure conform to a U-shaped relation in the east, as well as the central and western areas. Besides, the inflection point values are 0.263 and 0.247 separately, both of which are at the place where the U-shaped curve is dropping. The findings are identical to those of the full sample. The existing regulatory intensity is not favorable to optimize and upgrade the employment skill structure. Only by strengthening the regulation intensity and crossing the turning point as quickly as possible can command-and-control regulation fully play to the upgrading of the employment skill structure.

**Table 7:** Heterogeneous analysis for eastern, central and western China.

Variable	Eastern China			Central and Western China		
	(1)	(2)	(3)	(4)	(5)	(6)
ems1_i,t-1_	0.969***	4.732***	5.568***	0.812***	0.816***	0.826***
	(0.075)	(0.0000)	(0.0000)	(0.028)	(0.027)	(0.078)
CER_i,t_	-0.132*			-0.204**		
	(0.069)			(0.084)		
CER_i,t_^2^	0.251***			0.413***		
	(0.064)			(0.121)		
MER_i,t_		1.181***			-0.125***	
		(0.121)			(0.016)	
MER_i,t_^2^		-0.465***			0.125***	
		(0.174)			(0.035)	
VER_i,t_			0.743***			0.072***
			(0.035)			(0.005)
VER_i,t_^2^			-2.069***			0.308
			(0.022)			(0.313)
fdi_it_	-0.024	-0.796***	-0.779***	0.140***	0.140***	0.857***
	(0.039)	(0.005)	(0.0004)	(0.043)	(0.049)	(0.036)
edu_it_	0.211	-4.413***	1.655***	1.046**	1.826*	4.191***
	(0.996)	(0.288)	(0.013)	(0.462)	(0.983)	(0.257)
k_it_	0.002	-0.099***	-0.133***	-0.0006	-0.0009	-0.001**
	(0.003)	(0.003)	(0.002)	(0.002)	(0.002)	(0.0006)
R&D_it_	0.0003	0.115***	0.152***	0.0007	0.0005	0.002***
	(0.003)	(0.002)	(0.001)	(0.0007)	(0.0008)	(0.0004)
lnwage_it_	0.047	-1.132***	-1.093***	0.043***	0.043***	0.122***
	(0.046)	(0.004)	(0.017)	(0.008)	(0.008)	(0.009)
Observations	209	209	209	361	361	361

Notes: Robust t–statistics are in parentheses

***, **, and * refer to the significance levels of 1%, 5% and 10%, respectively.

According to column (5) of Table 7, market-incentive type and job skill structure have a U-shaped connection in the middle and western regions. Its inflection point value is 0.5, and the current regulatory intensity is 0.139, which falls inside the negative impact interval of the curve, and the results is consistent with the whole sample. In order to surpass the inflection point and optimize the job skill structure, government should increase the regulatory intensity by continuously improving the emission trading system and augmenting the subsidies for green technologies.

However, we can see the column (2) in Table 7, the primary term of MER is significantly positive, while that of the secondary term is considerably negative in eastern. Market-incentive regulation and employment skill structure present an inverse U-shaped relation, which contradicts the results of the whole sample. Its turning point value is 1.27, and the current intensity is in the rising phase of the curve. In the eastern region, market-incentive regulation will considerably optimize the job structure at present.

This finding is similar to Zheng that environmental regulations excluded more workers in the central and western areas [18]. That might be because, for one thing, the eastern region’s economy is advanced, and the government has increased R&D subsidies for enterprises, which stimulates technological innovation of companies and increases the need of high-skilled talents. For another, in comparison to the central and western districts, eastern district has numerous universities and scientific research institutions with more high-skilled workers and lower labor costs. Faced with the imposition of environmental taxes, businesses will measure the tax expenses and human resource costs. Considering a long-term benefit, they will enhance R&D efforts and innovation, which will attract massive high- skilled workers. And the favorable employment environment in the east enables the labor market to adjust rapidly, providing opportunities for highly skilled workers. The innovation compensation impact and factor substitution effect are adequate to compensate the cost effect in this stage, so the current strength of market-incentive type optimizes eastern employment skill structure. However, stricter environmental regulations are not always better. For example, India has introduced a lot of water pollution regulations, but the Ganges is still seriously polluted [106]. If the intensity is too high, it will be detrimental to the existence of enterprises, and even lead to production reductions and layoffs [107], which reduces the demand for high-skilled workers. Therefore, eastern areas should control the strength of market-incentive type reasonably and attempt to keep it within the positive effect interval.

From column (6) in Table 7, voluntary environmental regulation is positively related to the employment skill structure at the 1% level, and there is no nonlinear relation. This indicates that increasing the strength of voluntary type will upgrade central and western areas’ job skill structure, that is correspond with the whole sample. This original study conclusion prompts the related provinces to facilitate the intensity of voluntary type by constantly enhancing citizens’ awareness of environmental protection and giving full play to citizens’ main role in environmental governance and other ways. For the eastern region, as shown in column (3), the elementary term and quadratic term of VER are notably 0.743 and -2.069, indicating that voluntary regulation and employment skill structure have an inverse U-shaped relationship. In addition, the current intensity has not yet surpassed the turning point. We discover that the finding in eastern is significant different. This because eastern area’ economy is flouring, citizens are more conscious of the need of environmental protection [106], and there are numerous channels for environmental supervision and complaints, so the intensity of it is higher. This has a clear deterrent effect on enterprises that they have to transform towards a greener and low-carbon future. As a result, the current increase in the intensity of voluntary type will facilitate the upgrading of the employment skill structure in the eastern region. However, it should be recognized that excessively stringent environmental regulation would reduce productivity [107]. And enterprises will pass on the costs to workers [49], reducing the demand for production employees and indirectly obstructing the optimization of employment skill structure. Therefore, rather than increasing the regulatory intensity arbitrarily, the eastern provinces should enhance their control on its intensity. The findings of the heterogeneity analysis not only expand the existing research but also have an obvious practical significance by allowing policymakers to enhance the strength of three kinds regulation in accordance with regional circumstances.

### 4.3 Mediating effect analysis

There are few literatures have revealed the relation among environmental regulation, industrial greening transition, and job skill structure. According to the disparate influences of environmental regulations upon the employment skill structure, this study first attempts to include industrial greening transition as the mediating variable in the analysis.

From Table 6, different kinds of environmental regulations and employment skill structure have a prominent relation, indicating that the mediation test can be performed. Table 8 displays the mediation results. According to the procedure of mediation test, the significance of coefficients (β_2_) and (γ_4_) should be tested on the premise that the coefficients (α_2_) and (α_3_) are significant. From column (1), CER’s elementary and square terms are notably positive and negative severally, which indicates that it appears an inverse U-typed impact that first facilitates and then restrains the industrial greening transition. According to column (4), the influence coefficient of industrial green transformation upon employment skill constitution is not significant.

**Table 8:** Results of the mediation test.

Variable	ingt_it_			ems1_i,t-1_		
	(1)	(2)	(3)	(4)	(5)	(6)
CER_i,t_	0.144***			-0.313***		
	(0.053)			(0.046)		
CER_i,t_^2^	-0.286**			0.541***		
	(0.127)			(0.100)		
MER_i,t_		0.115***			-0.172***	
		(0.012)			(0.019)	
MER_i,t_^2^		-0.137***			0.204***	
		(0.038)			(0.041)	
VER_i,t_			0.020			0.079***
			(0.051)			(0.022)
VER_i,t_^2^			-0.158			-0.007
			(0.121)			(0.057)
ingt_it_				0.003	0.018	-0.011
				(0.008)	(0.015)	(0.010)
fdi_it_	0.020*	-0.010	0.067***	-0.042***	-0.028**	-0.049***
	(0.011)	(0.007)	(0.015)	(0.009)	(0.013)	(0.017)
edu_it_	1.342*	0.413	1.287***	-0.421*	0.776***	1.267***
	(0.805)	(0.566)	(0.463)	(0.222)	(0.233)	(0.199)
k_it_	0.0006	0.0006	0.00007	0.004***	0.004***	0.005***
	(0.0006)	(0.0007)	(0.0009)	(0.0008)	(0.0007)	(0.0008)
R&D_it_	-0.00002	0.0003	0.0005	-0.0002	-0.0003	-0.0003
	(0.0006)	(0.0007)	(0.0006)	(0.0004)	(0.0004)	(0.0003)
lnwage_it_	-0.013	-0.003	-0.012	0.035***	0.009	-0.0006
	(0.011)	(0.008)	(0.010)	(0.007)	(0.009)	(0.008)
ingt_i,t−1_	0.825***	0.863***	0.808***			
	(0.037)	(0.035)	(0.033)			
ems1_i,t-1_				0.892***	0.942***	0.952***
				(0.012)	(0.014)	(0.010)
Constant	0.205*	0.099	0.220**	-0.305***	-0.108	-0.045
	(0.112)	(0.083)	(0.104)	(0.064)	(0.081)	(0.071)
P-AR(1)	0.0002	0.0002	0.0002	0.0002	0.0002	0.0002
P-AR(2)	0.0677	0.0753	0.0718	0.5360	0.9033	0.9971
P-Sargan	0.8275	0.8295	1.0000	1.0000	1.0000	0.9955
Observations	570	570	570	570	570	570

Notes: Robust t–statistics are in parentheses

***, **, and * refer to the significance levels of 1%, 5% and 10%, respectively.

Therefore, referring to Mac et al. [108], the research adopts the Bootstrap approach to investigate the estimated value (β_2_*γ_4_) of indirect effect. Table 9 displays the results of sampling 1000 times using the bootstrap test. The 95% confidence interval for indirect effect is [0.0084, 0.0412], meaning that its indirect effect value (β_2_*γ_4_) through industrial green transformation has a 95% probability to fall within the interval, which excludes 0. Hence, the indirect effect is prominently plus, while the direct impact is prominently minus. In light of MacKinnon’ research [88] about how to estimate whether it’s a mediating effect or a suppression effect, we find the symbols of indirect and direct effects are different. As a result, the industrial green transformation presents a "suppression effect". How to explain this phenomenon?

**Table 9:** Results of bootstrap test.

Environmental regulation	Indirect effect	Direct effect	95% Conf. Interval
Command-and-control type	0.0230***	-0.2084***	[0.0084, 0.0412]
Market-incentive type	0.0322***	0.0496*	[0.0129, 0.0561]
Voluntary type	-0.0519***	0.1245***	[-0.0759, -0.0346]

Notes: ***, **, and * refer to the significance levels of 1%, 5% and 10%, respectively.

Firstly, from Table 6 columns (1) and (2), command-and-control type and employment skill constitution present a U-type relation. Neither the mean regulation intensity within the sample period nor the average regulation intensity in 2019 have exceeded the inflection point, and the current intensity is in the descending phase of the curve. So, it will be detrimental to the upgrading of employment skill structure at present. That’s why the direct effect is negative. Further, command-and-control regulation and industrial greening transition present an inverse U-type relation. The current regulatory intensity is beneficial to promoting industrial green transformation. And the indirect effect is significantly plus in Table 9.

This is similar to the results of Li that environmental regulation will affect the activities of dirty industries as well as the employment [22]. This is because, for one thing, the execution of mandatory orders will force the polluting enterprises to shut down and relocate, lowing the percentage of polluting enterprises in the local area and the need of workers. And the surviving corporations will pursue technological innovation and upgrade environmental protection equipment, which enhances the resource utilization rate and supports the industrial greening transition.

Due to industrial activity and labor demand have a positive relationship, the green transformation will optimize and upgrade several conventional industries, raising a number of green industries that require highly talents [30]. This will optimize the employment skill constitution. Namely, the optimization effect of command-and-control environmental regulation on employment skill structure through industrial green transformation obscures the adverse direct impact of its present regulation intensity on the employment skill structure.

From column (2) of Table 8, MER’s elementary term and square term are significantly plus and minus respectively, demonstrating that market-incentive regulation shows an inverse U-shaped relation with industrial green transformation. Besides, the current regulatory intension is in the increasing phase of the curve, which implies that the implementation of it will accelerate industrial greening transition at this stage. In column (5), the effect coefficient (γ_4_) of industrial green transformation on employment skill structure is not significant, so we use Bootstrap method for sampling. From Table 9, the 95% confidence interval of market-incentive type does not include 0, and its indirect and direct effects are prominently plus. The symbols for indirect effect and direct effect are the same, indicating that the industrial green transformation acts a partly mediation effect between market-incentive environmental regulation and employment skill structure, with the mediating effect accounting for 27.83%.

This is because market-incentive regulation, including emission charges, environmental taxes, and R&D subsidies can effectively stimulate businesses to employ R&D on green technology and provide a strong intrinsic motivation for enterprises to apply cleaner production technologies. Some large enterprises with sufficient capitals will take the lead in traditional manufacturing transformation and new technology research [33], while small and medium-size enterprises will be driven to promote industrial green transformation collaboratively. A large number of R&D talents are needed throughout the transformation, which raises the need of high-skilled worker and further upgrades the employment skill structure. Hypothesis H4b is validated.

From columns (3) and (6) in Table 8, the effect of voluntary environmental regulation on industrial greening transition is insignificant. After Bootstrap test, it is found that the indirect effect of it is significantly negative from Table 9, and the 95% confidence interval excludes 0. In addition, the direct impact is prominently plus, showing that the industrial greening transition has a suppression effect between voluntary type and employment skill structure. This is inconsistent with hypothesis H4c. This may be because Chinese voluntary regulation is launched late and remains in the early phase of its development. For most provinces, the regulatory strength of it is quite low [109]. Despite public demand for environmentally friendly products has attracted the attention of industrial enterprises, but due to the fact that it is not a mandatory law or regulation, in contrast, it stems from public and enterprises’ environmental awareness and responsibility.

As a result, some industrial businesses absence the motivation of technical innovation when voluntary regulation’s strength is poor, preferring to pay fines and other less severe penalties, which decreases the needs of highly skilled employees and hinders the upgrade of employment skill structure [6]. Moreover, when faced with environmental administrative penalties brought by the public tip-offs, industrial enterprises usually pass on the costs of this violation to employees through salary reductions and layoffs, which is ultimately adverse to the optimization of job skill constitution. This means that, because of the low regulatory intensity of voluntary type at present, its negative impact on the employment skill structure through industrial green transformation obscures the direct optimization effect of it on the employment skill structure.

### 4.4 Robustness test

Considering the possible non-randomness of the empirical results [110], this section tests the results’ robustness. The paper substitutes variables and changes research methods to examine the stability robustness.

#### 4.4.1 The robust test of basic regression

Firstly, referring to other methods on measuring the employment skill structure, this section takes the nature of the work as a standard to differentiate between high-skilled and unskilled labor. Workers in R&D and managerial roles are considered as high-skilled labor in this article, while others are termed low-skilled labor. Moreover, the article takes the percentage of R&D staffs to all workers in industrial businesses above the scale (ems2_it_) as a proxy variable for robustness test. Secondly, given the possibility of randomness in the regression results using a single estimation method, and in order to embody the hysteresis effect of the employment skill structure, this section uses another estimation method for dynamic panel data, the Difference Generalized Method of Moment (DIF-GMM) [111]. Although it is not as efficient as system-GMM, this method can give some reference for the research results. Table 10 shows the robustness test results.

**Table 10:** The robustness test of basic regression.

Variable	ems_2it_			DIF-GMM		
	(1)	(2)	(3)	(4)	(5)	(6)
ems2_i,t-1_	0.313***	0.182***	0.169***	0.868***	0.900***	0.894***
	(0.089)	(0.061)	(0.054)	(0.018)	(0.009)	(0.018)
CER_i,t_	-0.138**			-0.259***		
	(0.062)			(0.058)		
CER_i,t_^2^	0.284**			0.478***		
	(0.124)			(0.126)		
MER_i,t_		-0.063***			-0.0142***	
		(0.012)			(0.010)	
MER_i,t_^2^		0.199***			0.144***	
		(0.055)			(0.017)	
VER_i,t_			0.070***			0.084**
			(0.021)			(0.033)
VER_i,t_^2^			-0.093			-0.017
			(0.075)			(0.050)
Control	Yes	Yes	Yes	Yes	Yes	Yes
Constant	-0.002	0.005	0.068	-0.736***	-0.628***	-0.621***
	(0.046)	(0.057)	(0.049)	(0.069)	(0.069)	(0.062)
P-AR(1)	0.0001	0.0001	0.0001	0.0002	0.0002	0.0002
P-AR(2)	0.8330	0.3506	0.3627	0.5500	0.9727	0.9310
P-Sargan	1.0000	0.9022	0.8890	1.0000	1.0000	1.0000
Observations	570	570	570	570	570	570

Notes: Robust t–statistics are in parentheses

***, **, and * refer to the significance levels of 1%, 5% and 10%, respectively.

Both command-and-control and market-incentive regulations have a U-shaped relation with employment skill structure whether the article replaces indicators or estimation method; while voluntary type is positively related to the employment skill structure, and there is no nonlinear relationship between them. These conclusions are generally the same as the basic regression, which confirms the results’ robustness.

#### 4.4.2 The robust test of mediating effect model

This section re-estimates the results using the DIF-GMM to validate the mediating effect results’ robustness in Table 11.

**Table 11:** Results of the mediation test.

Variable	ingt_it_			ems1_i,t-1_		
	Model (1)	Model (2)	Model (3)	Model (4)	Model (5)	Model (6)
CER_i,t_	0.145**			-0.331***		
	(0.058)			(0.056)		
CER_i,t_^2^	-0.374**			0.663***		
	(0.157)			(0.116)		
MER_i,t_		0.062**			-0.176***	
		(0.029)			(0.018)	
MER_i,t_^2^		0.097**			0.184***	
		(0.038)			(0.045)	
VER_i,t_			-0.097			0.097***
			(0.064)			(0.034)
VER_i,t_^2^			0.125			-0.016
			(0.166)			(0.048)
ingt_it_				0.004	0.053***	0.016
				(0.013)	(0.020)	(0.015)
ingt_i,t−1_	0.716***	0.637***	0.721***			
	(0.034)	(0.038)	(0.043)			
ems1_i,t-1_				0.856***	0.891***	0.890***
				(0.044)	(0.015)	(0.013)
Control	Yes	Yes	Yes	Yes	Yes	Yes
Constant	0.172	0.393***	0.202**	-0.697***	-0.617***	-0.637***
	(0.124)	(0.119)	(0.099)	(0.085)	(0.097)	(0.063)
P-AR(1)	0.0002	0.0001	0.0002	0.0003	0.0002	0.0002
P-AR(2)	0.0822	0.0743	0.1141	0.5748	0.9471	0.9731
P-Sargan	1.000	0.9978	1.0000	1.0000	1.0000	1.0000
Observations	540	540	540	540	540	540

Notes: Robust t–statistics are in parentheses

***, **, and * refer to the significance levels of 1%, 5% and 10%, respectively.

From Table 11, this paper discovers that whether we use DIF-GMM or system-GMM, the symbol and significance of the coefficients are generally identical with regression results. Besides, from column (5), the influence of industrial green transformation upon employment skill structure is significantly positive. That is, industrial green transformation acts as a partial mediator between MER and employment skill structure, confirming the results’ robustness. Columns (4) and (6) indicate the effects of industrial green transformation on employment skill structure are not significant for command-and-control and market-incentive types, which requires the Bootstrap test. It is the same as the regression findings of Table 8, which ensures the robustness of the conclusions.

## 5. Conclusions and suggestions

Using industrial green transition as a mediating variable, the article innovatively explores the heterogenous influences of diverse categories of environmental regulations upon the employment skill structure, which contributes to current researches. First of all, according to the distinctions in regulatory tools and effects, we classify regulations into three kinds and measure their regulatory strength respectively. Then this article explores the influence of three kinds of environmental regulation upon employment skill structure and analyzes regional heterogeneity. Moreover, the article is the first attempt to integrate environmental regulation, industrial green transformation, and job skill structure into the same analytical frame, and explores the mediating role of industrial greening transition. Following are the findings:

(1) From a national perspective, both command-and-control and market-incentive types present a U-typed relation with job skill structure. The current regulatory intensity is in the declining phase of the curve, which has not yet crossed the inflection point. In a short time, increased the intensity of them will be detrimental to optimize and upgrade the job skill structure; but after crossing the inflection point in a long run, increasing the regulatory intensity will contribute to optimizing the employment skill structure. Hence, governments should enhance the intension of the two types regulation at present. Only by enduring the "pain" and surpassing the turning point will China be able to fully release potential function of them in optimizing the employment skill structure. Voluntary environmental regulation is positively correlated with employment skill structure, and they do not have a nonlinear relation. Increased intensity of it will upgrade the employment skill structure.

(2) The influences of diverse environmental regulation upon job skill structure are heterogenous. In central and western regions, the relation among the three regulations and employment skill structure is identical to the total sample. Command-and-control type and employment skill structure show a U-shaped relation in eastern areas, which is similar to the whole sample; nevertheless, market-incentive and voluntary type both show an inverse U-shaped relation with the employment skill structure, and their current intensity is falling inside the curve’s positive interval. So, the eastern government should strive to maintain regulatory strength at the rising stage of the curve.

(3) Market-incentive environmental regulation could upgrade employment skill structure via industrial green transformation. As a mediating variable, industrial green transformation plays a partial intermediary role between market-incentive type and employment skill structure. In contrast, for command-and-control type, industrial green transformation shows a suppression effect. Besides, the indirect and direct effects are prominently plus and minus respectively, which indicates that the indirect effect of it in optimizing the employment skill structure through industry green transformation obscures its adverse direct impact of the current intensity on the employment skill structure. Similarly, industrial green transformation also shows a suppression effect between voluntary type and employment skill structure. The immediate and indirect impacts are plus and minus severally, suggesting that the negative impact of voluntary type upon employment skill structure through industrial green transformation obscures its direct optimizing effect on employment skill structure.

(4) Finally, the trend of temporal changes and spatial distribution of the three environmental regulations are heterogeneous. As far as time, their intensity shows a tendency of raising, then decreasing and finally stabilizing from 2000 to 2019. The intensities of command-and-control and market-incentive regulations steadily decline, while the intensity of voluntary type gradually increases. At the same time, the degree of industrial greening transition has been increasing, and the geographical gaps have been diminishing. In terms of space, there are considerable discrepancies among eastern, central, and western regions. The eastern regulatory intensity is much greater than other areas. And the level of industrial greening transition decreases gradually from east to west, and regional imbalance still persists.

According to the above conclusions, in order to optimize and upgrade the employment skill structure, the governments ought to coordinate diverse tools of environmental regulations. First, the strength of command-and-control type ought to be properly increased, and the governments should fulfill its principal function in formulating and implementing environmental policies. Second, the governments are supposed to use market-incentive type exactly, continuously further the emission trading system, and firmly enforce punitive measures such as environmental taxes. Meanwhile, enterprises should make full use of the government’s ecological transfer payment funds to stimulate labors in innovating. However, for the eastern provinces, the government should grasp the regulation intensity of market-incentive type and endeavor to keep it in the upward stage of the curve, instead of arbitrarily enhancing the regulation strength. In addition, China should increase the intensity of voluntary type gradually. The governments ought to propagandize the environmental protection policies, raise civic consciousness on environmental ownership in order to encourage citizens to actively participate in pro-environment. Finally, governments should deepen the positive role of market-incentive regulation on industrial green transformation and accelerate industrial green transformation by strictly controlling capacity inventory, accelerating industrial structure upgrade, and cultivating emerging industries and other ways, so that the partial mediating role of industrial green transformation between market-incentive type and employment skill structure is fully realized.

Furthermore, this article takes provincial data as the research sample, which reduces the difference of the effect between cities to some extent. As a result, in the follow-up research, we will continue to refine the data and integrate urban data to more properly evaluate the relation between environmental regulations, industrial green transformation, and employment skill structure.

## Supporting information

S1 Table The data of all variables. (XLSX)

S1 Appendix The web address of data sources. (DOCX)

## References

[1] Yang X, Xu J, Zhu M, Yang Y. Environmental regulation and corporate tax avoidance-Evidence from China. PLoS One. 2022;17(1):e0261037. Epub 2022/01/14. doi: 10.1371/journal.pone.0261037 ; PubMed Central PMCID: PMC8757985. 35025907 PMC8757985

[2] Li RYM, editor A review on 10 countries’ sustainable housing policies to combat climate change. 18th Annual Pacific-Rim Real Estate Society Conference, Adelaide, Australia; 2012.

[3] He Q, Han Y, Wang L. The impact of environmental regulation on green total factor productivity: An empirical analysis. PLoS One. 2021;16(11):e0259356. Epub 2021/11/02. doi: 10.1371/journal.pone.0259356 ; PubMed Central PMCID: PMC8559937. 34723997 PMC8559937

[4] Curtis EM. Who Loses under Cap-and-Trade Programs? The Labor Market Effects of the NOx Budget Trading Program. The Review of Economics and Statistics. 2018;100(1):151–66. doi: 10.1162/REST_a_00680

[5] Walker WR. The Transitional Costs of Sectoral Reallocation: Evidence From the Clean Air Act and the Workforce*. The Quarterly Journal of Economics. 2013;128(4):1787–835. doi: 10.1093/qje/qjt022

[6] Zhong S, Xiong Y, Xiang G. Environmental regulation benefits for whom? Heterogeneous effects of the intensity of the environmental regulation on employment in China. J Environ Manage. 2021;281:111877. Epub 2020/12/29. doi: 10.1016/j.jenvman.2020.111877 . 33370676

[7] Liu M, Tan R, Zhang B. The costs of “blue sky”: Environmental regulation, technology upgrading, and labor demand in China. Journal of Development Economics. 2021;150:102610.

[8] Zhang H, Liu Z, Zhang Y-J. Assessing the economic and environmental effects of environmental regulation in China: The dynamic and spatial perspectives. Journal of Cleaner Production. 2022;334:130256.

[9] Dissou Y, Sun Q. GHG Mitigation Policies and Employment: A CGE Analysis with Wage Rigidity and Application to Canada. Canadian Public Policy. 2013;39(Supplement 2):S53–S65. doi: 10.3138/CPP.39.Supplement2.S53

[10] Nunes P, Pinheiro F, Brito MC. The effects of environmental transport policies on the environment, economy and employment in Portugal. Journal of cleaner production. 2019;213:428–39.

[11] Li M, Du W. The impact of environmental regulation on the employment of enterprises: an empirical analysis based on scale and structure effects. Environ Sci Pollut Res Int. 2022;29(15):21705–16. Epub 2021/11/14. doi: 10.1007/s11356-021-17460-z . 34773235

[12] Wang Y, Yang Y, Fu C, Fan Z, Zhou X. Environmental regulation, environmental responsibility, and green technology innovation: Empirical research from China. PLoS One. 2021;16(9):e0257670. Epub 2021/09/23. doi: 10.1371/journal.pone.0257670 ; PubMed Central PMCID: PMC8457501. 34551024 PMC8457501

[13] Pollin R, Garrett-Peltier H, Heintz J, Hendricks B. Green growth a US program for controlling climate change and expanding job opportunities. 2014.

[14] Coglianese J, Gerarden TD, Stock JH. The Effects of Fuel Prices, Environmental Regulations, and Other Factors on U.S. Coal Production, 2008–2016. The Energy Journal. 2020;41(1). doi: 10.5547/01956574.41.1.jcog

[15] Song M, Xie Q, Wang S, Zhou L. Intensity of environmental regulation and environmentally biased technology in the employment market. Omega. 2021;100:102201.

[16] Hafstead MAC, Williams RC. Unemployment and environmental regulation in general equilibrium. Journal of Public Economics. 2018;160:50–65. doi: 10.1016/j.jpubeco.2018.01.013

[17] Li Y, Hu M, Zhao L. Study on the impact of industrial green development and technological innovation on employment structure. Frontiers in Earth Science. 2023;11:1115476.

[18] Zheng J, He J, Shao X, Liu W. The employment effects of environmental regulation: Evidence from eleventh five-year plan in China. J Environ Manage. 2022;316:115197. Epub 2022/05/10. doi: 10.1016/j.jenvman.2022.115197 . 35533596

[19] Wang F. The intermediary and threshold effect of green innovation in the impact of environmental regulation on economic Growth: Evidence from China. Ecological Indicators. 2023;153:110371.

[20] Wang Y, Sun X, Guo X. Environmental regulation and green productivity growth: Empirical evidence on the Porter Hypothesis from OECD industrial sectors. Energy Policy. 2019;132:611–9. doi: 10.1016/j.enpol.2019.06.016

[21] Wang L, Wang Z, Ma Y. Heterogeneous environmental regulation and industrial structure upgrading: Evidence from China. Environmental Science and Pollution Research. 2022;29(9):13369–85. doi: 10.1007/s11356-021-16591-7 34591249

[22] Li Z, Lin B. Analyzing the impact of environmental regulation on labor demand: A quasi-experiment from Clean Air Action in China. Environmental Impact Assessment Review. 2022;93. doi: 10.1016/j.eiar.2021.106721

[23] Rubashkina Y, Galeotti M, Verdolini E. Environmental regulation and competitiveness: Empirical evidence on the Porter Hypothesis from European manufacturing sectors. Energy Policy. 2015;83:288–300. doi: 10.1016/j.enpol.2015.02.014

[24] Rassier DG, Earnhart D. Effects of environmental regulation on actual and expected profitability. Ecological Economics. 2015;112:129–40. doi: 10.1016/j.ecolecon.2015.02.011

[25] Wang Q, Su B, Zhou P, Chiu C-R. Measuring total-factor CO2 emission performance and technology gaps using a non-radial directional distance function: A modified approach. Energy Economics. 2016;56:475–82. doi: 10.1016/j.eneco.2016.04.005

[26] Zhou X, Xia M, Zhang T, Du J. Energy-and environment-biased technological progress induced by different types of environmental regulations in China. Sustainability. 2020;12(18):7486.

[27] Nowak MJ, Cotella G, Śleszyński P. The Legal, Administrative and Managing Framework for Spatial Policy, Planning and Land-Use. Interdependence, Barriers and Directions of Change: MDPI-Multidisciplinary Digital Publishing Institute; 2021.

[28] Duan Y, Jiang X. Pollution haven or pollution halo? A Re-evaluation on the role of multinational enterprises in global CO2 emissions. Energy Economics. 2021;97:105181.

[29] Raff Z, Earnhart D. The effects of Clean Water Act enforcement on environmental employment. Resource and Energy Economics. 2019;57:1–17. doi: 10.1016/j.reseneeco.2018.12.002

[30] Pinderhughes R. Green-collar jobs: An analysis of the capacity of green businesses to provide high quality jobs for men and women with barriers to employment: City of Berkeley Office of Energy and Sustainable Development; 2007.

[31] Dai L, Mu X, Lee CC, Liu W. The impact of outward foreign direct investment on green innovation: the threshold effect of environmental regulation. Environ Sci Pollut Res Int. 2021;28(26):34868–84. Epub 2021/03/05. doi: 10.1007/s11356-021-12930-w . 33660182

[32] Song Y, Yang T, Zhang M. Research on the impact of environmental regulation on enterprise technology innovation-an empirical analysis based on Chinese provincial panel data. Environ Sci Pollut Res Int. 2019;26(21):21835–48. Epub 2019/05/28. doi: 10.1007/s11356-019-05532-0 . 31134544

[33] Sun X, Zheng Y, Zhang C, Li X, Wang B. The Effect of China’s Pilot Low-Carbon City Initiative on Enterprise Labor Structure. Frontiers in Energy Research. 2022;9. doi: 10.3389/fenrg.2021.821677

[34] Wang X, Shao Q. Non-linear effects of heterogeneous environmental regulations on green growth in G20 countries: Evidence from panel threshold regression. Sci Total Environ. 2019;660:1346–54. Epub 2019/02/13. doi: 10.1016/j.scitotenv.2019.01.094 . 30743929

[35] Ren S, Li X, Yuan B, Li D, Chen X. The effects of three types of environmental regulation on eco-efficiency: A cross-region analysis in China. Journal of Cleaner Production. 2018;173:245–55. doi: 10.1016/j.jclepro.2016.08.113

[36] Xie R-h, Yuan Y-j, Huang J-j. Different Types of Environmental Regulations and Heterogeneous Influence on “Green” Productivity: Evidence from China. Ecological Economics. 2017;132:104–12. doi: 10.1016/j.ecolecon.2016.10.019

[37] Zheng D, Shi M. Multiple environmental policies and pollution haven hypothesis: Evidence from China’s polluting industries. Journal of Cleaner Production. 2017;141:295–304. doi: 10.1016/j.jclepro.2016.09.091

[38] Sun J, Zhai N, Miao J, Mu H, Li W. How do heterogeneous environmental regulations affect the sustainable development of marine green economy? Empirical evidence from China’s coastal areas. Ocean & Coastal Management. 2023;232:106448.

[39] Jiang X, Lu W-x, Zhao H-q, Yang Q-c, Chen M. Quantitative evaluation of mining geo-environmental quality in Northeast China: comprehensive index method and support vector machine models. Environmental Earth Sciences. 2014;73(12):7945–55. doi: 10.1007/s12665-014-3953-7

[40] Cao X, Deng M, Song F, Zhong S, Zhu J. Direct and moderating effects of environmental regulation intensity on enterprise technological innovation: The case of China. PLoS One. 2019;14(10):e0223175. Epub 2019/10/08. doi: 10.1371/journal.pone.0223175 ; PubMed Central PMCID: PMC6779245. 31589643 PMC6779245

[41] Langpap C, Shimshack JP. Private citizen suits and public enforcement: Substitutes or complements? Journal of Environmental Economics and Management. 2010;59(3):235–49. doi: 10.1016/j.jeem.2010.01.001

[42] Jiang Z, Wang Z, Lan X. How environmental regulations affect corporate innovation? The coupling mechanism of mandatory rules and voluntary management. Technology in Society. 2021;65:101575.

[43] Zhang Y, Song Y, Zou H. Non-linear effects of heterogeneous environmental regulations on industrial relocation: Do compliance costs work? Journal of Environmental Management. 2022;323:116188. doi: 10.1016/j.jenvman.2022.116188 36113295

[44] Mehmood S, Zaman K, Khan S, Ali Z. The role of green industrial transformation in mitigating carbon emissions: Exploring the channels of technological innovation and environmental regulation. Energy and Built Environment. 2023.

[45] Shen N, Liao H, Deng R, Wang Q. Different types of environmental regulations and the heterogeneous influence on the environmental total factor productivity: Empirical analysis of China’s industry. Journal of Cleaner Production. 2019;211:171–84. doi: 10.1016/j.jclepro.2018.11.170

[46] Ge Y, Zhi Q. Literature review: The green economy, clean energy policy and employment. Energy Procedia. 2016;88:257–64.

[47] Rogers KR, Pleasants R. Greening Community Colleges: An Environmental Path to Improving Educational Outcomes. Jobs for the Future. 2011.

[48] Albrizio S, Kozluk T, Zipperer V. Environmental policies and productivity growth: Evidence across industries and firms. Journal of Environmental Economics and Management. 2017;81:209–26. doi: 10.1016/j.jeem.2016.06.002

[49] Mishra V, Smyth R. Environmental regulation and wages in China. Journal of Environmental Planning and Management. 2012;55(8):1075–93. doi: 10.1080/09640568.2011.636556

[50] Porter ME, van der Linde C. Toward a New Conception of the Environment-Competitiveness Relationship. The Journal of Economic Perspectives. 1995;9(4):97–118.

[51] Liu Y, Wang J. Environmental pollution, environmental regulation, and labor income share. Environ Sci Pollut Res Int. 2020;27(36):45161–74. Epub 2020/08/12. doi: 10.1007/s11356-020-10408-9 . 32779069

[52] Guo W, Dai H, Liu X. Impact of different types of environmental regulation on employment scale: an analysis based on perspective of provincial heterogeneity. Environ Sci Pollut Res Int. 2020;27(36):45699–711. Epub 2020/08/18. doi: 10.1007/s11356-020-10428-5 . 32803588

[53] Li RYM, Li HCY. The challenge of sustainability in China’s built environment: a comparison between urban and rural areas. International Journal of Sustainable Real Estate and Construction Economics. 2018;1(2):123–41.

[54] Zhu J, Xu X. Available Energy and Environmental Economics. 2023.

[55] Wu W, Liu Y, Wu C-H, Tsai S-B. An empirical study on government direct environmental regulation and heterogeneous innovation investment. Journal of Cleaner Production. 2020;254. doi: 10.1016/j.jclepro.2020.120079

[56] Sun Z, Wang X, Liang C, Cao F, Wang L. The impact of heterogeneous environmental regulation on innovation of high-tech enterprises in China: mediating and interaction effect. Environ Sci Pollut Res Int. 2021;28(7):8323–36. Epub 2020/10/17. doi: 10.1007/s11356-020-11225-w . 33063211

[57] Liu B, Wang J, Li RYM, Peng L, Mi L. Achieving carbon neutrality-The role of heterogeneous environmental regulations on urban green innovation. Frontiers in Ecology and Evolution. 2022:471.

[58] Zhao X, Zhao Y, Zeng S, Zhang S. Corporate behavior and competitiveness: impact of environmental regulation on Chinese firms. Journal of Cleaner Production. 2015;86:311–22. doi: 10.1016/j.jclepro.2014.08.074

[59] Féres J, Reynaud A. Assessing the Impact of Formal and Informal Regulations on Environmental and Economic Performance of Brazilian Manufacturing Firms. Environmental and Resource Economics. 2011;52(1):65–85. doi: 10.1007/s10640-011-9520-8

[60] Xie W, Yan T, Xia S, Chen F. Innovation or Introduction? The Impact of Technological Progress Sources on Industrial Green Transformation of Resource-Based Cities in China. Frontiers in Energy Research. 2020;8. doi: 10.3389/fenrg.2020.598141

[61] Li R, Ramanathan R. Exploring the relationships between different types of environmental regulations and environmental performance: Evidence from China. Journal of Cleaner Production. 2018;196:1329–40. doi: 10.1016/j.jclepro.2018.06.132

[62] Zhang G, Jia Y, Su B, Xiu J. Environmental regulation, economic development and air pollution in the cities of China: Spatial econometric analysis based on policy scoring and satellite data. Journal of Cleaner Production. 2021;328. doi: 10.1016/j.jclepro.2021.129496

[63] Pan Z, Wang Z, Li X, Li J, Zhou Y. Space-Time Pattern of Coupling Coordination between Environmental Regulation and Green Water Resource Efficiency in China. Sustainability. 2022;14(17):10742.

[64] Lu W, Wu H, Geng S. Heterogeneity and threshold effects of environmental regulation on health expenditure: Considering the mediating role of environmental pollution. J Environ Manage. 2021;297:113276. Epub 2021/07/23. doi: 10.1016/j.jenvman.2021.113276 . 34293674

[65] Cui S, Wang Y, Zhu Z, Zhu Z, Yu C. The impact of heterogeneous environmental regulation on the energy eco-efficiency of China’s energy-mineral cities. Journal of Cleaner Production. 2022;350. doi: 10.1016/j.jclepro.2022.131553

[66] Wu C, Hua Y. Does Environmental Regulation Have an Employment Dividend? Evidence from China. Sustainability. 2023;15(7):6307.

[67] Feng C, Wang M, Liu G-C, Huang J-B. Green development performance and its influencing factors: A global perspective. Journal of Cleaner Production. 2017;144:323–33. doi: 10.1016/j.jclepro.2017.01.005

[68] Yang S, Bai Y, Wang S, Feng N. Evaluating the transformation of China’s industrial development mode during 2000–2009. Renewable and Sustainable Energy Reviews. 2013;20:585–94. doi: 10.1016/j.rser.2012.12.034

[69] Hou J, Teo TSH, Zhou F, Lim MK, Chen H. Does industrial green transformation successfully facilitate a decrease in carbon intensity in China? An environmental regulation perspective. Journal of Cleaner Production. 2018;184:1060–71. doi: 10.1016/j.jclepro.2018.02.311

[70] Du K, Cheng Y, Yao X. Environmental regulation, green technology innovation, and industrial structure upgrading: The road to the green transformation of Chinese cities. Energy Economics. 2021;98. doi: 10.1016/j.eneco.2021.105247

[71] Fu J, Xiao G, Wu C. Urban green transformation in Northeast China: A comparative study with Jiangsu, Zhejiang and Guangdong provinces. Journal of Cleaner Production. 2020;273. doi: 10.1016/j.jclepro.2020.122551

[72] Chen J. Study on the carbon emission reduction performance of resource tax reform: based on the perspective of substitution of factors of production. Open journal of business and management. 2016;5(1):182–93.

[73] Niu D, Wang Z, Yang S. Are environmental regulation tools effective? An analysis based on financial investment of entity enterprises. Frontiers in Environmental Science. 2022:2061.

[74] Wu Z, Liao JY, Wang S, Ji J, Zhao X. Corporate Leadership Strategy Management Based on Entropy Coupling Algorithm. Mobile Information Systems. 2021;2021:1–10.

[75] Wu M, Wu J, Zang C. A comprehensive evaluation of the eco-carrying capacity and green economy in the Guangdong-Hong Kong-Macao Greater Bay Area, China. Journal of Cleaner Production. 2021;281:124945.

[76] Pan X, Cheng W, Gao Y, Balezentis T, Shen Z. Is environmental regulation effective in promoting the quantity and quality of green innovation? Environ Sci Pollut Res Int. 2021;28(5):6232–41. Epub 2020/09/30. doi: 10.1007/s11356-020-10984-w . 32989701

[77] Goldsmith RW. A perpetual inventory of national wealth. Studies in Income and Wealth, Volume 14: NBER; 1951. p. 5–73.

[78] Wang D. The Linkage Mechanism between Environment-Related Rules and Environment-Related Efficiency of Industries in China: An Analysis Based on the Adaptive Semi-Parametric Panel Model. Sustainability. 2021;13(11):6203.

[79] Liang Q, Asuka J. A multidimensional energy poverty measurement in China-Based on the entropy method. Energy for Sustainable Development. 2022;71:554–67.

[80] Hiç Ö. Evolution of New Keynesian Economics. Procedia Computer Science. 2019;158:1025–32.

[81] Greenwald B, Stiglitz J. New and old Keynesians. Journal of Economic Perspectives. 1993;7(1):23–44.

[82] Arellano M, Bover O. Another look at the instrumental variable estimation of error-components models. Journal of econometrics. 1995;68(1):29–51.

[83] Blundell R, Bond S. Initial conditions and moment restrictions in dynamic panel data models. Journal of econometrics. 1998;87(1):115–43.

[84] Sun H, Chen F. The impact of green finance on China’s regional energy consumption structure based on system GMM. Resources Policy. 2022;76:102588.

[85] Zhou H, Xu G. Research on the impact of green finance on China’s regional ecological development based on system GMM model. Resources Policy. 2022;75. doi: 10.1016/j.resourpol.2021.102454

[86] Pociovălișteanu DM, Novo-Corti I, Aceleanu MI, Șerban AC, Grecu E. Employment policies for a green economy at the European Union level. Sustainability. 2015;7(7):9231–50.

[87] Judd CM, Kenny DA. Process analysis: Estimating mediation in treatment evaluations. Evaluation review. 1981;5(5):602–19.

[88] Baron RM, Kenny DA. The moderator-mediator variable distinction in social psychological research: conceptual, strategic, and statistical considerations. J Pers Soc Psychol. 1986;51(6):1173–82. Epub 1986/12/01. doi: 10.1037//0022-3514.51.6.1173 . 3806354

[89] MacKinnon DP, Krull JL, Lockwood CM. Equivalence of the mediation, confounding and suppression effect. Prev Sci. 2000;1(4):173–81. Epub 2001/08/29. doi: 10.1023/a:1026595011371 ; PubMed Central PMCID: PMC2819361. 11523746 PMC2819361

[90] Gan T, Liang W, Yang H, Liao X. The effect of Economic Development on haze pollution (PM2.5) based on a spatial perspective: Urbanization as a mediating variable. Journal of Cleaner Production. 2020;266. doi: 10.1016/j.jclepro.2020.121880

[91] Preacher KJ, Hayes AF. SPSS and SAS procedures for estimating indirect effects in simple mediation models. Behavior research methods, instruments, & computers. 2004;36:717–31. doi: 10.3758/bf03206553 15641418

[92] Wen Z, Ye B. Analyses of mediating effects: the development of methods and models. Advances in psychological Science. 2014;22(5):731.

[93] Wu J, Chang I-S. Environmental Management in China: Policies and Institutions: Springer; 2020.

[94] Hao M, Lyv K, Li S, Hu W. How does environmental regulation affect firm innovation? Evidence based on corporate life cycle. 2021.

[95] Hu D, Wang Y, Huang J, Huang H. How do different innovation forms mediate the relationship between environmental regulation and performance? Journal of Cleaner Production. 2017;161:466–76. doi: 10.1016/j.jclepro.2017.05.152

[96] Liang H, Dong L, Luo X, Ren J, Zhang N, Gao Z, et al. Balancing regional industrial development: analysis on regional disparity of China’s industrial emissions and policy implications. Journal of Cleaner Production. 2016;126:223–35. doi: 10.1016/j.jclepro.2016.02.145

[97] Li RYM, Chau KW. Econometric analyses of international housing markets: Routledge; 2016.

[98] Levin A, Lin C-F, Chu C-SJ. Unit root tests in panel data: asymptotic and finite-sample properties. Journal of econometrics. 2002;108(1):1–24.

[99] Habib SS, Sharif MS, Hossain MA. Nexus between economic growth, tourism revenue and financial development in Bangladesh: A time series analysis. Business and Economic Research. 2019;9(3):134–49.

[100] Im KS, Pesaran MH, Shin Y. Testing for unit roots in heterogeneous panels. Journal of econometrics. 2003;115(1):53–74.

[101] Huang J, Xu X, Zhao T. The moderating effect of clean technology innovation in the process of environmental regulation affecting employment: Panel data analysis based on 22 industrial sectors in China. Journal of Cleaner Production. 2023:137672.

[102] Zhang B, Bi J, Yuan Z, Ge J, Liu B, Bu M. Why do firms engage in environmental management? An empirical study in China. Journal of Cleaner Production. 2008;16(10):1036–45. doi: 10.1016/j.jclepro.2007.06.016

[103] Zhang J, Wang J, Yang X, Ren S, Ran Q, Hao Y. Does local government competition aggravate haze pollution? A new perspective of factor market distortion. Socio-Economic Planning Sciences. 2021;76:100959.

[104] Bruno GSF. Estimation and Inference in Dynamic Unbalanced Panel-data Models with a Small Number of Individuals. The Stata Journal. 2005;5(4):473–500. doi: 10.1177/1536867x0500500401

[105] Böhringer C, Rutherford TF. The costs of compliance: a CGE assessment of Canada’s policy options under the Kyoto protocol. World Economy. 2010;33(2):177–211.

[106] Li RYM, Li YL, Crabbe MJC, Manta O, Shoaib M. The impact of sustainability awareness and moral values on environmental laws. Sustainability. 2021;13(11):5882.

[107] Raff Z, Earnhart D. Employment and environmental protection: The role of regulatory stringency. J Environ Manage. 2022;321:115896. Epub 2022/09/16. doi: 10.1016/j.jenvman.2022.115896 . 36104878

[108] Mackinnon DP, Lockwood CM, Williams J. Confidence Limits for the Indirect Effect: Distribution of the Product and Resampling Methods. Multivariate Behav Res. 2004;39(1):99. Epub 2004/01/01. doi: 10.1207/s15327906mbr3901_4 ; PubMed Central PMCID: PMC2821115. 20157642 PMC2821115

[109] Li C, Li G. Does environmental regulation reduce China’s haze pollution? An empirical analysis based on panel quantile regression. PLoS One. 2020;15(10):e0240723. Epub 2020/10/29. doi: 10.1371/journal.pone.0240723 ; PubMed Central PMCID: PMC7592848. 33112878 PMC7592848

[110] Lin Q, Luo X, Lin G, Yang T, Su W. Impact of relocation and reconstruction policies on the upgrading of urban industrial structure in old industrial districts. Frontiers in Environmental Science. 2022;10:1002993.

[111] Arellano M, Bond S. Some tests of specification for panel data: Monte Carlo evidence and an application to employment equations. The review of economic studies. 1991;58(2):277–97.

