# Supplementary material for: The effect of heterogeneous environmental regulations on the employment skill structure: The system-GMM approach and mediation model
Source: PLoS One. 2023 Aug 24;18(8):e0290276. doi: 10.1371/journal.pone.0290276 (PMC10449222; doi:10.1371/journal.pone.0290276)
Supplement: S1 Appendix — (DOCX) [file pone.0290276.s002.docx]

**The following are the web address of data resource:**

**National Bureau of Statistics:** <https://data.stats.gov.cn/>

**China National Knowledge Infrastructure:** <https://data.cnki.net/yearBook?type=type&code=A>

**Provincial government websites and statistical offices:**

<http://tj.nmg.gov.cn/>; <https://www.beijing.gov.cn/>; <https://www.shanghai.gov.cn/>; [http://tjj.fujian.gov.cn/xxgk/ndsj/; http://www.hubei.gov.cn/xxgk/](http://tjj.fujian.gov.cn/xxgk/ndsj/;%20http://www.hubei.gov.cn/xxgk/); <http://tjj.hubei.gov.cn/tjsj/>; <http://www.hainan.gov.cn/>; <https://www.yn.gov.cn/>; <http://tjj.qinghai.gov.cn/>; <https://www.ln.gov.cn/>; <https://stats.tj.gov.cn/>; <http://www.jiangsu.gov.cn/>; <http://www.jiangxi.gov.cn/>; <http://www.hunan.gov.cn/>; <http://www.cq.gov.cn/>; <https://tj.nx.gov.cn/>; <http://tjj.hebei.gov.cn/>; <http://tjj.jl.gov.cn/tjsj/>; <http://tjj.zj.gov.cn/>; <http://www.shandong.gov.cn/>; <http://stats.gd.gov.cn/>; <https://www.sc.gov.cn/>; <http://tjj.shaanxi.gov.cn/>; <http://tjj.xinjiang.gov.cn/;http://www.shanxi.gov.cn/>; <https://www.hlj.gov.cn/>; <http://tjj.ah.gov.cn/ssah/index.html>; <https://www.henan.gov.cn/>; <http://tjj.gxzf.gov.cn/>; <http://stjj.guizhou.gov.cn/>; <http://tjj.gansu.gov.cn/>”
